# Gene variants associated with acne vulgaris presentation and severity: a systematic review and meta-analysis

**DOI:** 10.1186/s12920-021-00953-8

**Published:** 2021-04-13

**Authors:** Anna Hwee Sing Heng, Yee-How Say, Yang Yie Sio, Yu Ting Ng, Fook Tim Chew

**Affiliations:** Allergy and Molecular Immunology Laboratory, Lee Hiok Kwee Functional Genomics Laboratories, Department of Biological Sciences, Faculty of Science, National University of Singapore, Block S2, Level 5, 14 Science Drive 4, Lower Kent Ridge Road, Singapore, 117543 Singapore

**Keywords:** Acne vulgaris, Gene, Genome-wide association study, Single nucleotide polymorphism, Risk factors

## Abstract

**Background:**

Multiple factors have been attributed to acne vulgaris predisposition and individual variations in the severity of skin symptoms, and genetics stood out as one of the major factors.

**Methods:**

We performed a systematic review on the genes and their variants that have been investigated for association with acne presentation and severity. A random-effect meta-analysis using the allele model (minor allele vs. major allele) was also conducted to provide an overall estimation of risk effects of frequently reported gene variants. This included a subset data of 982 acne cases and 846 controls extracted from our existing GWAS database on various allergic and skin diseases among Singapore Chinese.

**Results:**

Systematic review of 51 articles covering Asians and Caucasians found 60 genes/loci and their 100 variants implicated in acne; majority of them were in the intron, coding region/missense, and promoter regions. The commonly studied candidate genes/gene families include tumor necrosis factor (*TNF*), and the interleukin (*IL*) and cytochrome P450 (*CYP*) gene families. Our meta-analysis showed that most of the analyzed gene variants exhibited insignificant pooled odds ratio (pOR) and significant heterogeneity between studies. Nevertheless, we found that *TNF* rs1800629 A allele carriers and *CYP17A1* rs743572 T allele carriers had significantly reduced mild acne risk [pOR: 0.60; 95% Confidence Interval (CI): 0.33–0.86] and severe acne risk (pOR: 0.59; 95% CI: 0.40–0.79), respectively, across populations. Overall, *FST* (follistatin) rs629725 A allele poses a significantly modest increased risk for acne presentation (pOR: 1.19, 95% CI: 1.14, 1.23), but neither *TIMP2* (TIMP metallopeptidase inhibitor 2) rs8179090 nor *CYP1A1* rs4646903 (pOR: 0.96, 95% CI: 0.80–1.12; pOR: 0.95, 95% CI: 0.83, 1.08), respectively. We discovered 15 novel SNPs in the 3′ UTR region of the Toll-like Receptor 4 gene (*TLR4*) associated with acne presentation.

**Conclusions:**

This systematic review and meta-analysis suggest that genes influencing inflammatory responses, specifically *TNF*, and genes influencing the function and activity of sebaceous glands, specifically *CYP17A1* and *FST*, have potential risk variants for acne presentation and severity across populations. Understanding the genetic susceptibility factors and biological pathways involved in the pathogenesis of acne will help us to gain insights into developing effective acne treatments.

**Supplementary Information:**

The online version contains supplementary material available at 10.1186/s12920-021-00953-8.

## Background

Acne vulgaris (acne) is a highly prevalent, chronic inflammatory skin disease affecting the pilosebaceous unit, mainly at the face, neck, upper trunk and back [1]. The severity of acne is characterized by the number of non-inflammatory closed and open comedones, inflammatory pustules and papules, as well as residual pathology like nodules and cysts [1, 2]. Chronic acne inflammatory symptoms like scars, erythema and hyperpigmentation, lead to psycho-social consequences such as depression, anxiety [3] and unemployment [4].

The etiology of acne is a complex interplay between androgen-induced sebum production, follicular keratinization, inflammation, and colonization of pilosebaceous follicles by *Cutibacterium acnes* (formerly *Propionibacterium acnes*) [1]. Acne is a multifactorial disease, and we have recently reviewed that the epidemiological risk factors for acne and acne severity include demographics, genetics/hormonal, dietary habits and lifestyle factors [5]. Of note, earlier twin [6] and family studies [7, 8] have established that acne susceptibility has a strong genetic component. Multiple case–control familial studies and twin studies involving numerous ethnicities showed strong heritability with estimates upwards of 78% [6–8], while concordance between monozygotic twins compared with dizygotic twins was higher for both acne presentation and severity [8]. Recent candidate gene studies and genome-wide association studies (GWAS) further revealed that the genes and loci associated with acne presentation and severity influence the function and activity of sebaceous glands or immune and inflammatory responses (reviewed in 9,10). Two such gene susceptibility loci were found in Han Chinese [9], while up to 15 were found in Caucasians [10–12].

Reviews on the genes and gene variants associated with acne presentation and severity are scarce [13–15]. Therefore, we hereby performed a systematic review on that, and also performed a random-effect meta-analysis on selected risk gene variants, which included a subset data of 982 acne cases and 846 controls extracted from our existing GWAS database on various allergic and skin diseases among Singapore Chinese. Lastly, we discussed the biological pathways implicated by these genes in the pathogenesis of acne.

## Methods

### Literature search

The articles reviewed were retrieved from searches conducted on the Web of Science database on 19 October 2020. In the first search, the search criteria used were: document type ‘article’, search terms ‘acne’ in the topic and ‘polymorphism’ in the title. Additional searches using the following criteria—document type ‘article’ and either the search terms ‘acne’ and ‘predisposition’ in the topic or search terms ‘acne risk’ and ‘gene’ in the topic—were also conducted. The searches aimed to garner articles about polymorphisms associated with acne presentation (presence/absence of acne vulgaris, severe acne, or teenage acne) and acne severity (mild, moderate, severe grades), thus more general search terms were chosen. Monogenic disorders and syndromes associated with (severe) acne (such as Apert syndrome, Frank–ter Haar syndrome and Winchester syndrome), and genes and gene variants associated with co-morbidities of acne such as endocrine system syndromes were not included in the systematic review as they have been reviewed recently [15]. Quality of included studies was evaluated using the JBI Critical Appraisal Tool Checklist [16] containing eight criteria. All papers selected for inclusion in the systematic review (that is—those that meet the inclusion criteria described above) were subjected to rigorous appraisal by two critical appraisers.

### Criteria for meta-analysis

Studies were included in the meta-analysis if they satisfied the following inclusion criteria: 1. Gene variants that were involved in at least two case–control studies that evaluated acne presentation and/or acne severity; 2. Genotype/allele frequencies were available for cases and controls; 3. The distribution of genotypes in the control group fulfils the Hardy–Weinberg equilibrium (HWE); 4. Studies that have provided an estimation of effect size, such as the odds ratio (OR) with corresponding 95% confidence intervals (CI); or if not reported in article, derived from statistical analysis using genotype/allele frequencies or from communication with corresponding authors; 5. When publications involved the overlapping data sets, only the study with the largest number of participants was included. The supporting PRISMA checklist is available as Additional file 1: Table S1.

### Data extraction and retrieval

The following data were extracted: the name of the first author, year of publication, country of origin, ethnicity, genotype frequencies in acne cases and controls or acne severity grades, presence/absence of association, OR, CI and *p* values for genotypes and alleles. Additional information about the gene variants were retrieved from National Centre for Biotechnology Information (NCBI) dbSNP or European Bioinformatics Institute (EBI) Ensembl databases: chromosomal location, common name of variant, rs number (if available), most severe consequence. Availability of expression quantitative trait loci (eQTL) data for the SNPs and the tissue types was sourced from the GTex Portal (available at https://gtexportal.org/home/). Gene ontology analysis/network analysis was performed for the list of genes using the online Database for Annotation, Visualization and Integrated Discovery (DAVID) v6.8 software (available at https://david.ncifcrf.gov/), with the highest classification stringency and other default settings for functional annotation clustering.

A subset of genotyping data from our existing GWAS database on various allergic and skin diseases among Singapore Chinese were extracted for 982 acne cases and 846 controls (full details in Additional file 2). After exclusion, a total of 4517 SNPs were analyzed and logistic regression analysis was conducted using the plink software to investigate the association between the minor allele counts and acne presentation.

### Statistical analysis for meta-analysis

To perform the random-effect meta-analysis, we extracted the OR and 95% CI reported from each study of interest. Where not reported, OR and CI were calculated with binary logistic regression test using IBM® SPSS® Statistics software (IBM Inc., NY). For consistency, we estimated the association based on the allele model (minor allele *vs*. major allele). These study findings were combined using the random effect model and the pooled OR and 95% CI were computed using the Stata/MP® version 16.0 statistical software (StataCorp LLC, TX). We used a chi-square-based test to examine any heterogeneity presented in the pooled risk estimate, with the inconsistency index (I^2^) also computed; I^2^ ≥ 50%, *p* < 0.05 was considered statistically significant. To evaluate whether the association showed any ethnicity-specific or severity-specific effects, we analyzed the data for separate subgroups defined by ethnicity or acne severity grade. Sensitivity analysis was performed by excluding individual studies and recalculating the results in order to assess the stability of the results. It was performed only for SNPs with ≥ 4 studies as we have set the criteria that the meta-analysis should only be performed for ≥ 3 studies. Publication bias was assessed using Begg’s funnel plots and Egger’s test.

## Results

### Overview of systematic review and meta-analysis of included studies

**Fig. 1:**
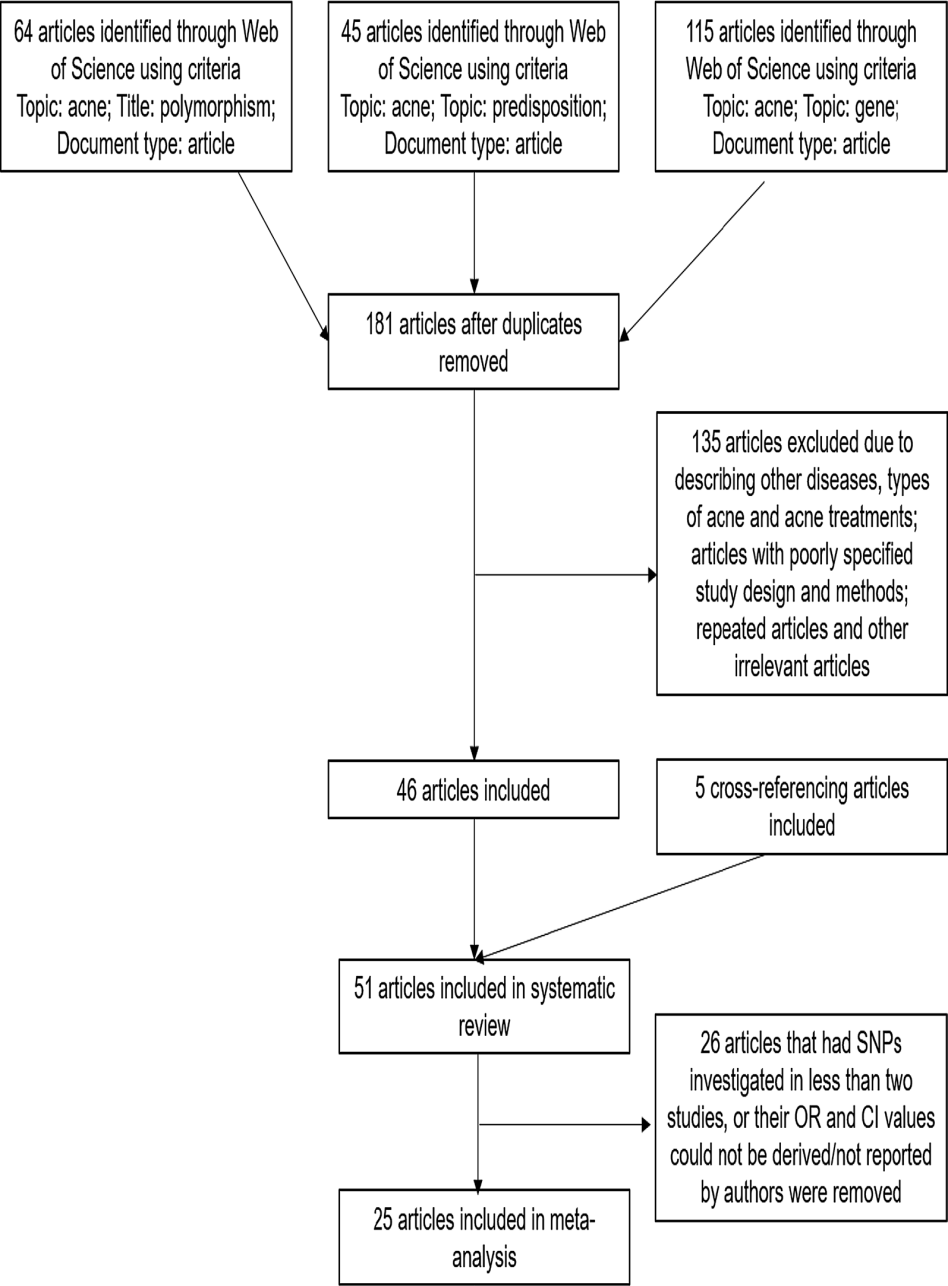
Flow chart of literature search and study selection

The flow chart that displays the study selection process is shown in Fig. 1. The three searches identified 64, 45 and 115 results respectively; however, there were overlaps in the results obtained from the three searches. After excluding articles describing other diseases, types of acne and acne treatments; articles with poorly specified study design and methods; repeated articles and other irrelevant articles, 46 unique articles remained. Five cross-referencing articles were also included, thus a total of 51 articles were included in the systematic review. Excluding 26 articles that had gene variants that were involved in less than two case–control studies (excluding ours) or had OR and CI values that could not be derived or not reported by authors, 25 articles were included in the meta-analysis.

Sixty genes and their 100 variants investigated in the reviewed articles and their associations with acne presentation and acne severity are summarized in Table 1. The most common region where the variants are located is the intron, followed by the coding region-missense, promoter, intergene, coding region—synonymous, 3′ UTR and non-coding exon region. Populations covered in the studies included Asians, Europeans and North Americans, but not Africans, South Americans and Oceanians. DAVID gene ontology analysis revealed 15 functional annotation clusters (Additional file 3: Figure S2). Most of the genes identified in the reviewed studies and from DAVID analysis can be categorized into two major biological function groups, namely ‘immune and inflammatory responses’ and ‘sebaceous gland function and activity’ (Table 1). Genes and gene variants from different populations studies and their differing association findings will be first described, with meta-analysis results and in vitro findings investigating the biological functional consequences of these SNPs included as well, where available.

**Table 1:** Genes/loci and their variants investigated in the reviewed articles and their association with acne presentation and severity

Gene	Chromosomal location^‡^	Common name of variant	rs number	Most severe consequence	eQTL availability for the associated gene in column 1 (from GTex Portal); tissue type	Phenotype	Association?	Population	Size of study (case/control)	Genotype/allele: OR (CI); *p* value	Authors, year of publication (Reference)
**Genes involved in inflammatory and immune responses**											
*ACE*	17q23	ID VNTR	–	Intron	No	Acne	Yes	Egypt	100/120	ID: 1.780 (1.086–2.918); 0.022 DD: 3.735 (2.289–6.097); < 0.001 D: 3.581 (2.376 5.397); < 0.001	Sorour et al. [17]
*IL1A*	2q14.1	− 889 C/T	rs1800587	Promoter	Yes; Nerve—Tibial, Spleen	Acne	Yes	Poland	115/100	TT: 3.77 (1.02–13.85); 0.044 T: 1.386 (0.909–2.112); 0.129	Sobjanek et al. [18]
						Acne	Yes	Pakistan	430/380	TT: 2.62 (1.98–3.5); < 0.0001 T: 2.012 (1.605–2.524); < 0.0001	Younis and Javed, [19]
						Acne	Yes	Greece	100/100	TT: 4.818 (1.959–11.847); < 0.001 T: 1.704 (1.134–2.561); < 0.01	Ibrahim et al. [20]
		+ 4845/ + 340 G/T (Ala114Ser)	rs17561	Coding region; missense	Yes; Spleen, Nerve—Tibial	Acne	Yes	Hungary and Romania	229/127	Not reported	Szabó et al. [21]
						Severe acne	No	USA	81/847	OR, CI not reported; p-value reported as “not significant”	Zhang et al. [12]
*IL1RN*	2q14.1	86 bp VNTR	–	Intron	No	Acne	No	Hungary and Romania	229/127	Not reported	Szabó et al. [21]
*IL1B*	2q14.1	− 511 C/T	rs16944	Intergene	Yes; Testis	Acne, acne severity and acne scarring	No	Turkey	90/30	Not reported	Akoglu et al. [22]
*IL4R*	16p12.1	A/G (Gln551Arg)	rs1801275	Coding region; missense	No	Acne	Yes	Saudi Arabia	95/93	GG (vs. GA + AA): 8.5 (3.6–20.4); < 0.001 G: 2.7 (1.7–4.2); < 0.001	Al Robaee et al. [23]
						Acne severity	No	Saudi Arabia	95/93	Not reported	Al Robaee et al. [23]
*IL4*	5q31.1	− 590 T/C	rs2243250	Promoter	Yes; Pituitary, Testis, Lung	Acne and acne severity	No	Saudi Arabia	95/93	Not reported	Al Robaee et al. [23]
*IL10*	1q32.1	− 1082 A/G	rs1800896	Promoter	No	Acne and acne severity	No	Saudi Arabia	166/390	Not reported	Al-Shobaili et al. [24]
*ITLN1*	1q23.3	+ 326 A/T (Val109Asp)	rs2274907	Coding region; missense	Yes; Minor Salivary Gland, Testis, Whole Blood	Acne	No	Turkey	65/44	Val/Asp: 0.696 (0.310–1.564); 0.381 Val/Val: 1.839 (0.442–7.653); 0.403 Val/Asp + Val/Val: 0.834 (0.386–1.800); 0.643	Soguktas, et al. [25]
*IL17A*	6p12.2	–	rs2275913	Intergene	No	Severe acne, Acne as teenager	No	USA	22,616/247,345	A: 1.0062 (0.9858,1.0271); 0.554 OR not available for acne as teenager phenotype	Ehm et al. [26]
*IL17F*	6p12.2	His161Arg	rs763780	Coding region; missense	No	Severe acne, Acne as teenager	No	USA	22,616/247,345	C: 1.0 (0.9548,1.0 74); 0.999 OR not available for acne as teenager phenotype	Ehm et al. [26]
*IL17RB*	3p21.1	Gln484Stop	rs1043261	Coding region; stop gained	Yes; Thyroid	Severe acne, Acne as teenager	No	USA	22,616/247,345	T: 0.9738 (0.9391,1.0098); 0.151 OR not available for acne as teenager phenotype	Ehm et al. [26]
*IL23R*	1p31.3	Arg381Gln	rs11209026	Coding region; missense	No	Severe acne, Acne as teenager	No	USA	22,616/247,345	A: 0.9790 (0.9414,1.0181); 0.288 OR not available for acne as teenager phenotype	Ehm et al. [26]
*MAPK11, OVOL1*	22q13.33	–	rs144991069	Intergene	No	Severe acne	Yes	UK	3,823/16,144	A: 1.85 (1.50–2.28); 1.01 × 10^−8^	Petridis et al. [11]
*MMP2*	16q12.2	− 1306 C/T	rs2285053	Promoter	No	Acne	No	Turkey	43/42	CT: 0.75 (0.3–1.86); 0.532 TT: 0.42 (0.06–2.91); 0.381 CT + TT: 0.7 (0.29–1.69); 0.427	Yaykasli et al. [27]
						Acne	Yes	China	251/121	CT: 0.285 (0.154–0.529); < 0.001 TT: 0.614 (0.559–0.674); 0.171 CT + TT: 0.275 (0.148–0.509); < 0.001 T: 0.306 (0.170–0.550); < 0.001	Gao et al. [28]
						Acne severity	No	China	251/121	Not reported	Gao et al. [28]
*NLRP3*	1q44	–	rs10754558	3′ UTR	No	Acne	Yes	China	428/384	G: 1.22 (1.00–1.48); < 0.05	Shen et al. [29]
		–	rs4612666	Intron	No	Acne	No	China	428/384	T: 1.05 (0.86–1.28); 0.64	Shen et al. [29]
*RETN*	19p13.2	− 420 C/G	rs1862513	Promoter	No	Acne and acne severity	Yes	Pakistan	180/180	G: 1.8 (1.23–2.89); 0.002	Hussain et al. [29]
						Acne and acne severity	Yes	Pakistan	530/550	G: 1.24 (1.05–1.47); 0.013	Younis et al. [30]
		+ 299 G/A	rs3745367	Intron	No	Acne and acne severity	Yes	Pakistan	530/550	A: 1.314 (1.10–1.57); 0.002	Younis et al. [30]
		− 420/299 haplotype	rs1862513, rs3745367	Promoter, intron	No	Acne	Yes	Pakistan	530/550	G–A: 1.61 (1.29–2.03); 0.0001	Younis et al. [30]
*SELL*	1q24.2	–	rs7531806	Intron	Yes; Brain—Putamen (basal ganglia), Brain—Caudate (basal ganglia), Brain—Cortex	Severe acne	Yes	China	GWAS: 1,056/1,056 Replication: 1,860/3,660 Combined: 2,916/4,716	A allele GWAS: 1.20 (1.06–1.36); 3.54 × 10^−3^ Replication: 1.23 (1.13–1.34); 9.22 × 10^−7^ Combined: 1.22 (1.12–1.28); 1.20 × 10^−8^	He et al. [9]
						Severe acne	No	UK	1,893/5132	A: 1.0292 (0.9852–1.0752); 0.197	Navarini et al. [10]
						Severe acne	No	UK	3,823/16,144	A: 1.03 (0.98–1.07); 0.206	Petridis et al. [11]
*SEMA4B*	15q26.1	–	rs34560261	Intron	Yes; Skin—Not Sun Exposed (Suprapubic, Skin—Sun Exposed (Lower leg), Esophagus—Mucosa	Severe acne	Yes	UK	3,823/16,144	C: 1.35 (1.24–1.47); 1.82 × 10^−12^	Petridis et al. [11]
		–	rs1533326	Intron	No	Severe acne	Yes	UK	3,823/16,144	A: 1.08 (1.03–1.13); 0.00308	Petridis et al. [11]
*TIMP2*	17q25.3	− 418 G/C	rs8179090	Promoter (transcription factor binding site)	No	Acne	No	Turkey	43/42	GC: 0.581 (0.21–1.58); 0.29 CC: 1.45 (0.23–8.87); 0.686 GC + CC: 0.7 (0.28–1.76); 0.451	Yaykasli et al. [27]
						Acne and acne severity	No	China	251/121	For acne only (severity not reported) GC: 1.220 (0.737–2.021); 0.439 CC: 2.082 (0.573–7.569); 0.256 GC + CC: 1.301 (0.802–2.110); 0.285 C: 1.327 (0.869–2.027); 0.189	Gao et al. [28]
*TLR2*	4q31.3	+ 2179 C/T or + 2029 C/T (Arg677Trp)	rs121917864	Coding region; missense	No	Acne	No	Hungary	63/38	SNP not detected	Koreck et al. [31]
		+ 2258 G/A (Arg753Gln)	rs5743708	Coding region; missense	No	Acne	No	Hungary	63/38	SNP not detected	Koreck et al. [31]
						Severe acne	No	USA	81/847	Not reported (not in dataset)	Zhang et al. [12]
						Acne and acne severity	Yes	China		Gln/Gln: 2.261 (1.052–4.858); 0.034	Tian et al. [32]
*TLR4*	9q33.1	+ 1063 A/G (Asp299Gly)	rs4986790	Coding region; missense	No	Acne	No	Hungary	63/38	Not reported	Koreck et al. [31]
						Severe acne	No	USA	81/847	Not reported	Zhang et al. [12]
		+ 1363 C/T (Thr399Ile)	rs4986791	Coding region; missense	No	Acne	No	Hungary	63/38	Not reported	Koreck et al. [31]
						Severe acne	No	USA	81/847	Not reported	Zhang et al. [12]
		–	15 SNPs (full list in Additional file 2)	3′ UTR	Please refer to Additional file 2: Table S2.2	Acne	Yes	Singapore	982/846	Please refer to Additional file 2: Table S2.2	Our unpublished data, 2020
*TNF*	6p21.33	− 238 G/A	rs361525	Promoter	No	Acne and acne severity	Yes	Pakistan	140/160	A: 1.6 (1.06–2.44); < 0.03 GA + AA Mild: 0.677 (0.353–1.3); 0.241 Moderate: 2.00 (0.798–5.015); 0.139 Severe: 2.678 (1.405–5.104); 0.003	Aisha et al. [33]
						Acne	No	Poland	84/75	A: 1.358 (0.472–3.911);0.57	Sobjanek et al. [34]
						Acne and acne severity	No	Hungary and Romania	224/112	A: 1.29 (0.57–2.91); 0.539 GA + AA Mild, moderate, severe: OR could not be computed as GA + AA for controls is 0	Szabó et al. [35]
		− 308 G/A	rs1800629	Promoter	No	Acne and acne severity	Yes	Pakistan	140/160	A: 1.5 (1.07–2.19); 0.02 GA + AA Mild: 0.5 (0.278–0.901); 0.021 Moderate: 2.364 (1.094–5.105); 0.029 Severe: 10.333 (3.036–35.174); < 0.001	Aisha et al. [33]
						Acne, acne severity and acne scarring	No	Turkey	90/30	A: 0.547 (0.277–1.079);0.082 GA + AA Mild: 0.737 (0.233–2.332); 0.603 Moderate: 0.571 (0.175–1.865); 0.354 Severe: 0.643 (0.221–1.87); 0.417	Akoglu et al. [22]
						Acne	Yes	Saudi Arabia	166/390	0.879 (0.636–1.216);0.436	Al-Shobaili et al. [24]
						Acne severity	No	Saudi Arabia	166/390	GA + AA vs GG Mild:0.698 (0.328–1.488); 0.352 Moderate: 1.047(0.581–1.888);0.878 Severe: 1.257 (0.643–2.455); 0.504	Al-Shobaili et al. [24]
						Acne	Yes	Turkey	113/114	A: 4.138 (2.25–7.612); < 0.0001	Baz et al. [36]
						Acne severity	No	Turkey	113/114	GA + AA Mild: 6.6 (2.737–15.918); < 0.0001 Moderate: 3.3 (1.489–7.316); 0.003 Severe: 5.775 (2.349–14.199); < 0.0001	Baz et al. [36]
						Acne	No	Greece	185/165	A: 1.684 (0.914–3.102);0.095	Grech et al. [37]
						Acne	No	Poland	85/75	A: 0.616 (0.329–1.151);0.129	Sobjanek et al. [34]
						Acne	Yes (females); No (males, total population)	Hungary and Romania	224/112	A: 1.455 (0.935–2.265);0.097	Szabó et al. [35]
						Acne severity	No	Hungary and Romania	224/112	GA + AA Mild: 1.379 (0.569–3.341); 0.477 Moderate: 1.532 (0.907–2.589); 0.111 Severe: 1.585 (0.754–3.334); 0.225	Szabó et al. [35]
						Acne	No	USA	81/847	Not reported	Zhang et al. [12]
		− 857 C/T	rs1799724	Promoter	No	Acne and acne severity	Yes	Hungary and Romania	224/112	CT + TT: 1.79 (1.14–2.81); 0.010	Szabó et al. [35]
						Severe acne	No	USA	81/847	Not reported	Zhang et al. [12]
		− 863 C/A	rs1800630	Promoter	No	Acne and acne severity	No	Hungary and Romania	224/112	CA + AA: 0.93 (0.54–1.59); 0.781	Szabó et al. [35]
		− 1031 T/C	rs1799964	Promoter	No	Acne and acne severity	No	Hungary and Romania	224/112	TC + CC: 1 (0.61–1.63); 1	Szabó et al. [35]
		− 238 G/A, − 308 G/A, − 376 G/A haplotype	rs361525, rs1800629, rs1800750	Promoter	No	Acne	No	Greece	185/165	Not reported	Grech et al. [37]
*TNFRSF1B*	1p36.22	Met196Arg	rs1061622	Coding region; missense	No	Acne and acne severity	Yes	China	93/90	M/R + R/R: 1.899 (1.036–3.445); 0.037	Tian et al. [32]
						Severe acne	No	USA	81/847	Not reported	Zhang et al. [12]
*TRAF3IP2*	6q21	Asp10Asn	rs33980500	Coding region; synonymous	Yes; Cells—Cultured fibroblasts	Severe acne	Yes	USA	22,616/247,345	Asn: 0.93 (0.89,0.96); 0.000059	Ehm et al. [26]
						Acne as teenager	No	USA	22,616/247,345	OR not available for acne as teenager phenotype	Ehm et al. [26]
*TYK2*	19p13.2	Ile684Ser	rs12720356	Coding region; missense	Yes; Whole Blood, Adrenal Gland	Acne as teenager	Yes	USA	22,616/247,345	OR not available for acne as teenager phenotype; *p* = 0.000105	Ehm et al. [26]
**Genes involved in sebaceous gland function and activity**											
*ADH7*	4q23	–	rs1154469	Splice region	No	Severe acne	Yes	China	1,024/1,029	T: 1.111 (1.001–1.233) 4.85 × 10^−2^	Yang et al. [38]
*AR*	Xq12	CAG (Gln) repeat VNTR	–	Coding region; Exon 1	No	Acne	Yes	China	238/207	CAG < 23 Males: 2.07 (1.21–3.54); 0.008 Females: 2.05 (1.18–3.56); 0.013	Pang et al. [39]
						Acne and acne severity	Yes (males); No (females)	China	206/200	Genotype/allele frequency not reported Mean CAG repeat length in males: 22.07 ± 3.026 control vs 20.61 ± 2.423 cases; *p* < 0.001	Yang et al. [40]
						Acne	No (overall, males) Yes (female)	Turkey	100/99	Genotype/allele frequency not reported Mean CAG repeat length: case 19.34 (20.22 in males and 18.88 in females) *vs*. control 19.7 (19.18 for males and 19.96 for females); *p* > 0.05 for overall & *p* = 0.0059 among females	Demirkan et al. [41]
						Acne	No (both males & females)	USA mixed (Caucasians, African, Hispanic)	12/12	Genotype and allele frequency not reported Mean CAG repeat length: case 20 ± 3 *vs*. control 20 ± 3; *p* = 0.27	Sawaya and Shalita [42]
		GGN (Gly) repeat VNTR	–	Coding region; Exon 1	No	Acne	No	China	237/205	GGN ≤ 23 Males: 1.75 (0.72–4.26); 0.264 Females: 0.90 (0.39–2.08); 0.83	Pang et al. [39]
*CYP17A1*	10q24.32	− 34 T/C	rs743572	5′ UTR/Promoter	Yes; Thyroid, Adipose—Subcutaneous, Nerve—Tibial	Acne and acne severity	Yes	Iran	198/195	T Overall: 2.31 (1.47–3.64); < 0.001 Mild: 2.35 (1.37–4); 0.001 Moderate: 2.06 (1.08–3.92); 0.03 Severe: 2.52 (1.37–4.63); < 0.01	Chamaie-Nejad et al. [43]
						Severe acne	No	USA	81/847	Not reported	Zhang et al. [12]
						Acne and acne severity	Yes	Uzbekistan	165/97	T Overall: 3.14 (2.07–4.76); < 0.05 Mild: 0.788 (0.43–1.444); 0.44 Severe: 15.05 (8.014–28.27); < 0.05 Moderate: 3.4 (2.07–5.39); < 0.05	Malikova et al. [44]
						Acne	No (Males) Yes (Overall)	China	206/200	T: 0.722 (0.548–0.951); 0.021	He et al. [45]
						Acne severity	Yes (Males); No (females)	China	206/200	T Mild + Moderate: 0.931 (0.633–1.307);0.68 Severe: 0.562 (0.401–0.789); 0.001	He et al. [45]
*CYP19A1*	15q21.2	Trp39Arg	rs2236722	Coding region; missense	No	Acne and acne severity	Yes	Iran	198/195	C Overall: 2.03 (1–4.12); < 0.05 Mild: 3.0 (1.33–6.71); < 0.01	Chamaie-Nejad et al. [43]
			rs700518	Coding region; synonymous	Yes; Whole Blood, Cells—Cultured fibroblasts, Skin—Sun Exposed (Lower leg)	Acne and acne severity	Yes	Iran	181/144	A Overall: 1.71 (1.25–2.34); 0.001 Mild: 1.64 (1.1–2.43); 0.014 Moderate: 1.67 (1.05–2.66); 0.03 Severe: 1.6 (1.02–2.51); 0.042	Ebrahimi et al. [46]
*CYP1A1*	15q22–q24	+ 6235 T/C	rs4646903	Intergene	Yes; Brain—Nucleus accumbens (basal ganglia)	Acne	No	Indonesia	35/35	C: 0.738 (0.372–1.464);0.384	Darmani et al. [47]
						Acne	No	Germany	96/408	C: 1.21 (0.68–2.16); 0.52	Paraskevaidis et al. [48]
		+ 4889 A/G (Ile462Phe/Val/Leu)	rs1048943	Coding region; missense	No	Acne	No	Germany	96/408	G: 1.02 (0.41–2.52); 0.96	Paraskevaidis et al. [48]
*DDB2*	11p11.2	–	rs747650	Intron	Yes; Skin—Not Sun Exposed (Suprapubic), Skin—Sun Exposed (Lower leg), Esophagus—Mucosa, Adipose—Subcutaneous, Muscle—Skeletal, Heart—Atrial Appendage	Severe acne	Yes	China	GWAS: 1,056/1,056 Replication: 1,860/3,660 Combined: 2,916/4,716	G allele GWAS: 1.29 (1.14–1.47); 8.12 × 10^−5^ Replication: 1.22 (1.12–1.33); 9.25 × 10^−6^ Combined: 1.24 (1.16–1.34) 4.41 × 10^−9^	He et al. [9]
						Severe acne	No	UK	1,893/5,132	G: 0.9886 (0.945–1.0342); 0.6188	Navarini et al. [10]
						Severe acne	No	UK	3,823/16,144	G: 1.01 (0.97–1.06); 0.601	Petridis et al. [11]
		–	rs1060573	Intron	Yes; Skin—Not Sun Exposed (Suprapubic), Skin—Sun Exposed (Lower leg), Esophagus—Mucosa, Adipose—Subcutaneous, Muscle—Skeletal, Heart—Atrial Appendage	Severe acne	Yes	China	GWAS: 1,056/1,056 Replication: 1,860/3,660 Combined: 2,916/4,716	G allele GWAS: 1.29 (1.14–1.47); 9.00 × 10^−5^ Replication: 1.20 (1.10–1.31); 2.44 × 10^−5^ Combined: 1.23 (1.15–1.33); 1.28 × 10^−8^	He et al. [9]
*FST*	5q11.2	–	rs38055	Intergene	No	Severe acne	Yes	UK	Discovery: 1,893/5,132 2nd stage: 2,207/2,087	A allele Discovery: 1.17 (1.08–1.27); 6.02 × 10^−5^ 2nd Stage:1.24 (1.13–1.36); 1.03 × 10^−5^ P_meta_: 4.58 × 10^−9^	Navarini et al. [10]
						Severe acne	Yes	UK	3823/16,144	A: 1.19 (1.14–1.24); 1.57 × 10^−14^	Petridis et al. [11]
			rs629725	Promoter (transcription factor binding site)	No	Severe acne	Yes	UK	3823/16,144	T: 1.20 (1.14–1.27); 8.22 × 10^−12^	Petridis et al. [11]
*IGF1*	12q23.2	CA repeat VNTR	–	5′ UTR/Promoter	No	Acne	No	Egypt	50/50	192/192: 2.708 (0.668–10.983); 0.163	El-Tahlawi et al. [49]
						Acne severity	No	Egypt	50/50	Not reported	El-Tahlawi et al. [49]
						Acne and acne severity	Yes	India	80/80	192/192: 4.29 (1.38–13.33); 0.01	Rahaman et al. [50]
						Acne and acne severity	Yes	Turkey	115/117	192/192: 0.875 (0.336–2.281); 0.785	Tasli et al. [51]
*HSD11B1*	1q32.2	–	rs12086634	Intron	No	Acne	No	Egypt	50/50	A: 3.197 (0.839–12.18); 0.088	Farag et al. [52]
		–	rs846910	Intron	No	Acne	Yes	Egypt	50/50	G: 3.296 (1.61–6.748); 0.001	Farag et al. [52]
						Acne severity	No	Egypt	50/50	Not reported	Farag et al. [52]
*HSD3B1*	1p12	–	rs6428829	Intron	No	Acne	Yes	China	403/207	G: 1.963 (1.206–3.197); 0.006	Yang et al. [53]
		Ile79Val	rs6201	Missense	No	Acne	No	China	403/207	G: 0.938 (0.49–1.795) 0.846	Yang et al. [53]
		Leu338Val	rs6203	Missense	No	Acne	No	China	403/207	T: 0.92 (0.717–1.18) 0.511	Yang et al. [53]
		Ile79Val, Leu338Val, –	rs6201, rs6203, rs6428829 haplotype	Coding region; missense, Coding region; missense, Intron	No	Acne	Yes	China	403/207	AAT: 0.653 (0.627–0.681); 0.00001	Yang et al. [53]
*HSD17B3*	9q22.32	–	rs2257157	Intron	No	Acne	No	China	403/207	G: 0.874 (0.678–1.126) 0.297	Yang et al. [53]
		–	rs7039978	Intron	Yes; Nerve—Tibial, Adipose—Subcutaneous, Thyroid	Acne	No	China	403/207	G: 0.804 (0.626–1.033) 0.087	Yang et al. [53]
		–	rs2476923	Intron	Yes; Nerve—Tibial, Adipose—Subcutaneous, Thyroid	Acne	No	China	403/207	G: 0.867 (0.681–1.103) 0.245	Yang et al. [53]
		–	rs11788785	Intron	Yes; Adipose—Subcutaneous	Acne	No	China	403/207	G: 1.173 (0.924–1.489) 0.19	Yang et al. [53]
		Gly289Cys/Arg/Ser	rs2066479	Missense	Yes; Nerve—Tibial, Heart—Atrial Appendage	Acne	No	China	403/207	G: 1.086 (0.821–1.437) 0.562	Yang et al. [53]
		–	rs8190557	Intron	Yes; Nerve—Tibial, Heart—Atrial Appendage	Acne	No	China	403/207	G: 1.098 (0.829–1.456) 0.514	Yang et al. [53]
		–	rs10990258	Intron	Yes; Thyroid	Acne	No	China	403/207	G: 1.102 (0.848–1.433) 0.466	Yang et al. [53]
		–	rs11788083	Intron	No	Acne	No	China	403/207	G: 0.827 (0.641–1.066) 0.142	Yang et al. 2013 [53]
		–	rs8190504	Intron	Yes; Small Intestine—Terminal Ileum	Acne	No	China	403/207	G: 0.859 (0.645–1.145) 0.3	Yang et al. [53]
		–	rs2066476	5′ UTR	Yes; Small Intestine—Terminal Ileum	Acne	No	China	403/207	G: 0.997 (0.736–1.349) 0.983	Yang et al. [53]
		–	rs4743709	5′ UTR	Yes; Pancreas, Cells—Cultured fibroblasts, Colon—Transverse, Small Intestine—Terminal Ileum, Liver, Adipose—Visceral (Omentum), Artery—Tibial, Adipose—Subcutaneous, Skin—Not Sun Exposed (Suprapubic)	Acne	No	China	403/207	G: 0.929 (0.72–1.199) 0.572	Yang et al. [53]
		H8 haplotype	n/a	n/a	n/a	Acne	Yes	China	403/207	GGAAGGAAAA: 0.469 (0.296–0.744); 0.00185	Yang et al. [53]
*LAMC2*	1q25.3	–	rs10911268	Intergene	Yes; Nerve—Tibial, Brain—Cortex, Brain—Anterior cingulate cortex (BA24), Brain—Frontal Cortex (BA9), Adipose—Subcutaneous, Pituitary, Esophagus—Muscularis, Brain—Hypothalamus, Colon—Sigmoid, Heart—Atrial Appendage	Severe acne,	Yes	UK	3,823/16,144	C: 1.19 (1.13–1.25); 2.44 × 10^−10^	Petridis et al. [11]
*LGR6*	1q32.1	–	rs788790	Intergene	Yes; Testis, Heart—Atrial Appendage, Skin—Sun Exposed (Lower leg)	Severe acne	Yes	UK	3,823/16,144	C: 1.12 (1.06–1.17); 1.96 × 10^−5^	Petridis et al. [11]
*MUC1*	1q22	20 aa VNTR	–	Coding region	No	Severe acne	Yes	Japan	67/64	Not reported	Ando et al. [54]
*OVOL1*	11q13.1	–	rs478304	Promoter	Yes; Thyroid, Brain—Cerebellum, Brain—Cerebellar Hemisphere, Brain—Cortex	Severe acne	Yes	UK	Discovery: 1,893/5,132 2nd stage: 2,207/2,087	T allele Discovery: 1.20 (1.11–1.29); 9.58 × 10^−6^ 2nd stage: 1.26 (1.16–1.38); 2.65 × 10^−7^ Pmeta: 3.23 × 10^−11^	Navarini et al. [10]
						Severe acne	Yes	UK	3,823/16,144	T: 1.14 (1.09–1.19); 2.14 × 10^−9^	Petridis et al. [11]
		–	rs144991069	Intergene	No	Severe acne	Yes	UK	3,823/16,144	A: 1.85 (1.50–2.28); 1.01 × 10^−8^	Petridis et al. [11]
			rs61744384	Coding region; synonymous	No	Severe acne	Yes	UK	3,823/16,144	T: 1.16 (1.11–1.21); 2.95 × 10^−11^	Petridis et al. [11]
*PPARG*	3p25.2	P12A	rs1801282	Coding region; missense	Yes, Esophagus—Mucosa, Esophagus—Gastroesophageal Junction, Cells—EBV-transformed lymphocytes, Esophagus—Muscularis	Acne and acne severity	Yes	Egypt	100/100	G: 2.103 (1.262–3.503); 0.004	Amr et al. 2014 [55]
						Acne	Yes (for patients with age of acne onset ≥ 20 years); No (overall population)	Iran	198/195	G: 1.444 (0.911–2.287);0.118	Saeidi et al. [56]
						Acne severity	No	Iran	198/195	Not reported	Saeidi et al. [56]
		–	rs3856806	Coding region; synonymous	No	Acne and acne severity	No	Iran	198/195	Not reported	Saeidi et al. [56]
*SPECC1L*	22q11.23	–	rs28360612	Intron	Yes; Skin—Sun Exposed (Lower leg), Artery—Tibial, Artery—Aorta, Cells—Cultured fibroblasts, Muscle—Skeletal, Adipose—Subcutaneous, Skin—Not Sun Exposed (Suprapubic)	Severe acne	Yes	UK	3,823/16,144	T: 1.16 (1.09–1.23); 7.12 × 10^−7^	Petridis et al. [11]
*SRD5A2*	2p23.1	TA repeat VNTR	–	3′ UTR	No	Acne	Yes	China	49/50	L: 3.52 (1.73–7.16); < 0.005	Hu et al. [57]
		V89L	rs523349	Coding region; missense	No	Acne	No	China	49/50	Not reported	Hu et al. [57]
*VDR*	12q13.11	TaqI	rs731236	Coding region; synonymous	Yes; Testis	Acne	Yes	Egypt	30/30	Not reported	Swelam et al. [57]
		ApaI	rs7975232	Intron	Yes; Testis	Acne	Yes	Egypt	30/30	Not reported	Swelam et al. [58]
						Acne severity	No	Egypt	30/30	Not reported	Swelam et al. [58]
*WNT10A*	2q35	Phe228Ile	rs121908120	Coding region; missense	No	Severe acne	Yes	UK	3823/16,144	T: 2.10 (1.67–2.63); 1.40 × 10^−10^	Petridis et al. [11]
			rs72966077	Intron	No	Severe acne	Yes	UK	3,823/16,144	C: 1.33 (1.19–1.48); 4.23 × 10^−7^	Petridis et al. [11]
**Genes involved in both inflammatory and immune responses and sebaceous gland function and activity**											
*EGFR*	7p11.2	D994D	rs2293347	Coding region; synonymous	No	Acne	No	Turkey	156/154	T: 0.8092 (0.5049 –1.297); 0.3782	Aydingoz et al. [59]
		R521K	rs2227983	Coding region; missense	No	Acne	No	Turkey	156/154	A: 1.055 (0.7227–1.541); 0.7802	Aydingoz et al. [59]
			CA repeat intron 1 VNTR	Intron	No	Acne	No	Turkey	156/154	Genotype/allele frequency not reported CA repeat mean length: Acne 16.49 ± 1.769 *vs*. control 16.56 ± 1.844; *p* = 0.6818	Aydingoz et al. [59]
*IL6*	7p15.3	− 572 G/C	rs1800796	Promoter	No	Acne	Yes	Egypt	30/20	C: 5.44 (2.27–13.04); < 0.001	Ragab et al. [60]
						Acne severity	No	Egypt	30/20	Not reported	Ragab et al. [60]
						Acne	Yes	Pakistan	430/380	C: 2.011 (1.61–2.45); < 0.0001	Younis and Javed [19]
*IL8*	4q13.3	− 251 T/A	rs4073	Promoter	No	Acne and acne severity	Yes	Pakistan	264/264	A: 1.6 (1.16–2.19); 0.003	Hussain et al. [61]
						Acne	No	Poland	115/100	A: 1.074 (0.735–1.571); 0.711	Sobjanek et al. [18]
*TGFB2*	1q41	–	rs1159268	Promoter	Yes; Esophagus—Mucosa	Severe acne	Yes	UK	Discovery: 1,893/5,132 2nd stage: 2,207/2,087	A allele Discovery: 1.17 (1.08–1.26); 3.34 × 10^−6^ 2nd stage: 1.15 (1.05–1.26); 3.17 × 10^−3^ Pmeta: 4.08 × 10^−8^	Navarini et al. [10]
						Severe acne	Yes	UK	3,823/16,144	A: 1.14 (1.09–1.19); 4.74 × 10^−9^	Petridis et al. [11]
			rs1256580	Intron	No	Severe acne	Yes	UK	3,823/16,144	C: 1.23 (1.15–1.31); 1.12 × 10^−9^	Petridis et al. [11]
			rs6684868	Promoter (Tf binding site)	Yes; Esophagus—Mucosa	Severe acne	Yes	UK	3,823/16,144	A: 1.15 (1.10–1.20); 2.42 × 10^−10^	Petridis et al. [11]
			rs11118336	Intergene	No	Severe acne	Yes	UK	3,823/16,144	C: 1.11 (1.06–1.16); 1.49 × 10^−6^	Petridis et al. [11]
**Genes with other functions**											
*BCL11A*	2p16.1	–	rs2901000	Intergene	Yes; Esophagus—Mucosa	Severe acne	Yes	UK	3823/16,144	A: 1.16 (1.10–1.22); 2.53 × 10^−8^	Petridis et al. [11]
*F13A1*	6p25	Val34Leu	rs435048	Intron	No	Severe acne	Yes	China	1,024/1,029	1.371 (1.164–1.616); 1.54 × 10^−4^	Yang et al. [38]
*FGF2*	4q28.1	–	rs4487353	Intergene	Yes; Nerve—Tibial	Severe acne	Yes	UK	3,823/16,144	G: 1.13 (1.07–1.19); 3.83 × 10^−6^	Petridis et al. [11]
*GLI2*	2q14.2	–	rs1092479	Intergene	No	Severe acne	Yes	UK	3,823/16,144	C: 1.12 (1.06–1.19); 3.60 × 10^−5^	Petridis et al. [11]
*LINC00958*	38p12	–	rs2727365	Intron	Yes; Esophagus—Mucosa, Thyroid, Whole Blood, Skin—Not Sun Exposed (Suprapubic)	Severe acne	Yes	UK	3,823/16,144	G: 1.19 (1.13–1.25); 1.08 × 10^−10^	Petridis et al. [11]
*LOC105378977*	5q11.2	–	rs158639	Intron	No	Severe acne	Yes	UK	3,823/16,144	A: 1.15 (1.09–1.21); 9.22 × 10^−7^	Petridis et al. [11]
*RORC*	1q21.3	–	rs4845604	Intron	No	Severe acne, Acne as teenager	No	USA	22,616/247,345	A: 1.0023 (0.9740,1.0314); 0.876	Ehm et al. [26]
*PINX1*	8p23.1	–	rs28570522	Intron	No	Severe acne	Yes	UK	3,823/16,144	A: 1.14 (1.09–1.21); 4.206 × 10^−7^	Petridis et al. [11]
*SUGCT*	7p14.1	–	rs7809981	Intron	Yes; Skin—Sun Exposed (Lower leg)	Severe acne	Yes	UK	3,823/16,144	T: 1.15 (1.08–1.22); 3.74 × 10^−6^	Petridis et al. [11]
n/a	38p12	–	rs4133274	Non-coding exon	No	Severe acne	Yes	USA	81/847	G: 4.01 (2.37–6.82); 1.70 × 10^−6^	Zhang et al. [12]
n/a	38p12	–	rs13248513	Intergene	No	Severe acne	Yes	USA	81/847	C: 3.82 (2.29–6.36) 2.02 × 10^−6^	Zhang et al. [12]

Sensitivity analysis showed that the corresponding pooled ORs were not significantly altered by sequential omission of individual studies (data not shown). They remained significant or insignificant even after sequential omission of individual studies. The results of sensitivity analysis indicated the stability of our results. Begg’s funnel plot and Egger’s test showed that there was statistically significant publication bias for the meta-analysis of *TNF* rs1800629 acne presentation, mild acne and severe acne; *SELL* rs7531806; *PPARG* rs1801282; and *CYP17A1* rs743572 acne presentation, moderate acne and severe acne. However, the shape of the funnel plots for the rest of the SNPs analyzed seemed symmetrical and Egger’s test *p* value > 0.05, indicating that there were no evidences for obvious publication bias (Additional file 3: Figure S3).

### Genes and gene variants involved in immune and inflammatory responses

#### Tumor necrosis factor (TNF)

Some of the genes implicated in acne presentation and acne severity are involved in immune and inflammatory responses. The pro-inflammatory factor, tumor necrosis factor (*TNF*) is the most frequently studied gene in the reviewed articles. In particular, the studies investigated variations at positions − 238, − 308, − 857, − 863 and − 1031 localized in the promoter region of *TNF*, meaning that they may affect the gene expression positively or negatively, conferring a protective or detrimental effect on acne.

**Fig. 2:**
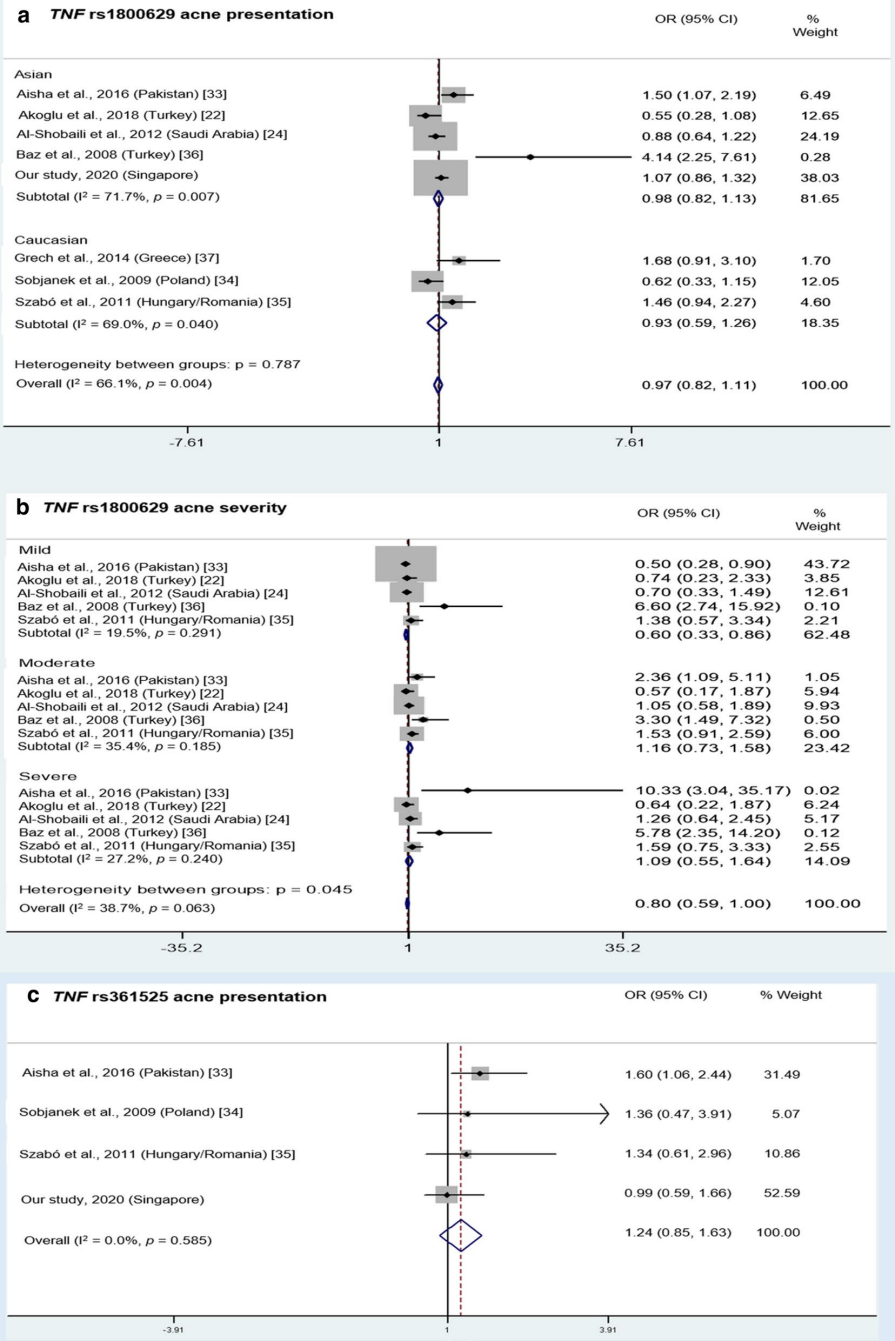
Meta-analysis of *TNF* SNPs associated with acne presentation and acne severity grades. **a** Subgroup meta-analysis of association of rs1800629 with acne presentation among Asians and Caucasians. **b** Subgroup meta-analysis of association of rs1800629 with acne severity among different grades. **c** Meta-analysis of association of rs361525 with acne presentation. Only gene variants that were investigated in at least two other previous studies were included in the meta-analysis. Analysis was performed under allele model (minor allele vs. major allele)., i.e. A versus G for both rs1800629 and rs361525

The − 308 SNP (rs1800629) is the most common among all and is a potential risk factor among Caucasians, as shown by two meta-analyses [37, 62], but the conclusions are different in other populations. Several studies found a significant association with acne alone [24, 36, 37] or both acne and acne severity [33], but other studies found insignificant associations with acne severity alone [24, 35, 36], acne alone [34] or both acne and acne severity [22]. Furthermore, the results of one study suggested the possibility of gender differences in the association of − 308 SNP with acne as a significant association was observed for females, but not for males or the combined population [35]. Meta-analysis of these and our study for acne presentation showed that the pooled OR was 0.97 (95% CI: 0.82–1.11) (Fig. 2a), while a subgroup analysis by ethnicity revealed an OR of 0.98 (95% CI: 0.82–1.13) and 0.93 (95% CI: 0.59–1.26) among Asians and Caucasians, respectively. Significant heterogeneity was observed across these reported findings, suggesting these analyzed findings had different study outcomes across each other (all I^2^ ≥ 50%, *p* < 0.05). This heterogeneity also corresponds to the publication bias detected for this SNP. While for acne severity, a subgroup analysis revealed an OR of 0.60 (95% CI: 0.33–0.86), 1.16 (95% CI: 0.73–1.58), and 1.09 (95% CI: 0.55–1.64) among mild, moderate and severe grades, respectively (Fig. 2b). No significant heterogeneity was found for all severity grades (I^2^ < 50%, *p* > 0.05), suggesting that rs1800629 A allele carriers had significantly reduced mild acne risk compared with G allele carriers across all populations.

Studies that investigated − 238 SNP (rs361525) showed mixed results. The − 238 SNP was significantly associated with acne and acne severity among Pakistanis [33], while no association with acne or with both acne and acne severity was reported among Caucasians in Poland [34] and Hungary/Romania [35]. Meta-analysis of these and our study showed that this SNP was not significantly associated with acne, as the pooled OR was 1.24 (95% CI: 0.85–1.63), with no significant heterogeneity observed (I^2^ = 0%, *p* = 0.585; Fig. 2c). Lastly, *TNF* − 238, − 308 and − 376 haplotypes did not show association with acne risk [37].

The − 857 SNP (rs1799724) was significantly associated with acne and acne severity among Caucasians [12, 35], while − 863 and − 1031 SNPs were not [34]. In the case of rs1799724, the major C allele exhibited a positive association with acne, whereas the minor T allele seemed to have a protective effect [35]; consistent with studies on other chronic inflammatory diseases [63]. The C/T base change generates a novel transcription factor binding site (OCT-1) at the promoter region of *TNF*, immediately next to a pre-existing NF-κB binding site, resulting in an altered *TNF* regulation in response to various stimuli [64]. This suggests the general importance of this SNP in regulating *TNF* expression.

In addition, two studies which investigated the M196R variation in *TNFR2* (Tumor Necrosis Factor Receptor 2) found a significant association with acne presentation and severity among Han Chinese [32], but not among Caucasians [12].

#### Interleukins (IL) and their associated antagonists and receptors

A frequently studied group of genes involved in inflammation and immune responses is the interleukins (IL) and their associated antagonists and receptors. The interleukin genes studied include *IL1A* (Interleukin-1α), *IL1B* (Interleukin-1β), *IL4* (Interleukin-4), *IL6* (Interleukin-6), *IL8* (Interleukin-8), *IL10* (Interleukin-10), *IL17A* (Interleukin-17A), *IL17F* (Interleukin-17F), while the antagonists and receptors include *IL1RN* (Interleukin 1 Receptor Antagonist) and *IL4R* (Interleukin-4 Receptor), *IL17RB* (Interleukin-17 Receptor B) and *IL23R* (Interleukin-23 Receptor).

**Fig. 3:**
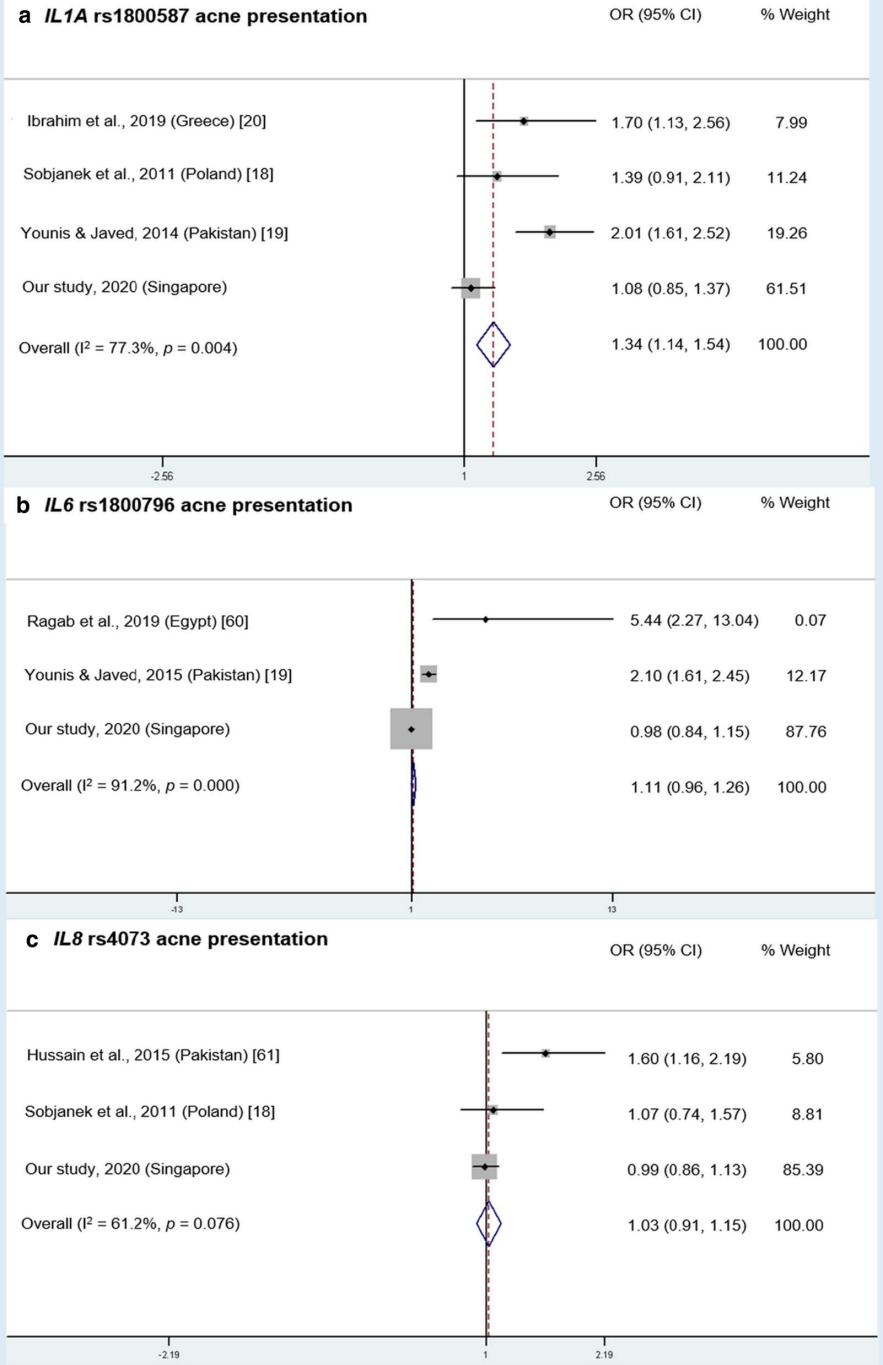
Meta-analysis of *IL* SNPs associated with acne presentation. **a** *IL1A* rs1800587. **b** *IL6* rs1800796. **c** *IL8* rs4073. Only gene variants that were investigated in at least two other previous studies were included in the meta-analysis. Analysis was performed under allele model (minor allele *vs*. major allele), i.e. T versus. C for rs1800587, C versus. G for rs1800796 and A versus T for rs4073

Several studies found that the *IL1A* − 889 C/T SNP (rs1800587) was associated with acne among Asian and European populations [18–20] while + 4845 G/T SNP only showed association among Caucasians in Hungary/Romania [21], but not in USA [12]. Meta-analysis of rs1800587 studies and our study showed that this SNP was significantly associated with acne, as the pooled OR was 1.34 (95% CI: 1.14–1.54), with significant heterogeneity observed (I^2^ = 77.3%, *p* = 0.004; Fig. 3a). Meanwhile, *IL1A* + 4845 G/T SNP causes an Ala114Ser substitution close to the proteolytic cleavage site of nuclear pre-IL-α (117/118) to mature IL-α, and might result in enhanced cleavage when the rare T allele is present [65, 66]. Pre-IL-1α has a predominantly nuclear localization, whereas mature IL-α exhibits a cytoplasmic localization and can be secreted in response to the appropriate signals [67]. Therefore, carriers of the T allele may have a higher risk of more severe acne symptoms that result from enhanced inflammatory reactions by increased IL-α secretion [13, 22].

In contrast, *IL1B* − 511 C/T, *IL4* − 590 T/C, *IL10* − 1082 A/G SNPs and *IL1RN* variable number tandem repeats (VNTR) all showed no significant association with acne and/or acne severity in the studied populations [21–24]. Furthermore, a study in Saudi Arabia found that the *IL4R* Q551R SNP was significantly associated with acne but not acne severity [23].

Two studies also found a significant association between *IL6* − 572 G/C variants and acne presentation [19, 60]. Meta-analysis of these and our study showed that this SNP was not significantly associated with acne, as the pooled OR was 1.11 (95% CI: 0.96–1.26), with significant heterogeneity observed (I^2^ = 91.2%, *p* = 0.000; Fig. 3b). Lastly, *IL8* − 251 T/A SNP was found to be significantly associated with acne and acne severity among Pakistanis [61], but not among Polish [18]; meta-analysis of these and our study showed that this SNP was not significantly associated with acne, as the pooled OR was 1.03 (95% CI: 0.91–1.15), with no significant heterogeneity observed (I^2^ = 61.2%, *p* = 0.076; Fig. 3c).

Lastly, Ehm et al. [26] found that *IL17A* rs2275913, *IL17F* rs763780, *IL17RB* rs1043261 and *IL23R* rs11209026 all had no significant association with both severe acne and teenage acne.

#### Toll-like receptor (TLR) family

Another gene family implicated is the toll-like receptor (*TLR*) gene family. None of the *TLR4* variants (rs4986790 and rs4986791) investigated in the reviewed articles were found to be associated with acne risk [12, 31]. Both of these SNPs change amino acids in the ligand recognition part of the receptor, but most in vitro functional studies suggest that the LPS-induced cytokine response of the Asp299Gly/Thr399Ile haplotype does not differ from that of the wild-type cytokine response [reviewed in [68]]. Nevertheless, there was a study which found increased tumor necrosis factor-α (TNF-α) cytokine response of the African-exclusive Asp299Gly/wild-type haplotype [69]. Using a subset of genotyping data of 982 acne cases and 846 controls extracted from our existing GWAS database (Additional file 2), we found that out of all the gene variants in this systematic review (Table 1), only *TLR4* (Additional file 2: Table S2.1) was significantly associated with acne presentation. Specifically, within 2 kb of this gene, 15 SNPs located in the 3′ UTR region (Additional file 2: Fig. S1), were significantly associated with acne presentation (*p* = 0.021222 for all SNPs; Additional file 2: Table S2.2).

Furthermore, no significant association with acne was found for two *TLR2* variants + 2179 C/T and + 2258 G/A among Caucasians [12, 31]. However, the latter SNP was significantly associated with acne and acne severity among Han Chinese [32]. The possible explanation in the discrepancy in the findings is that a highly homologous duplicated segment of exon 3 (harboring the SNP) exists 23 kb upstream of the *TLR2* locus [70]. In vitro data showed that the Arg to Gly change may impair TLR2-mediated immune signaling and the expression of various downstream target genes in response to microbial ligands [71, 72], suggesting that if this SNP may be a functional one in populations where it is truly present with reasonable frequency.

#### Other genes

**Fig. 4:**
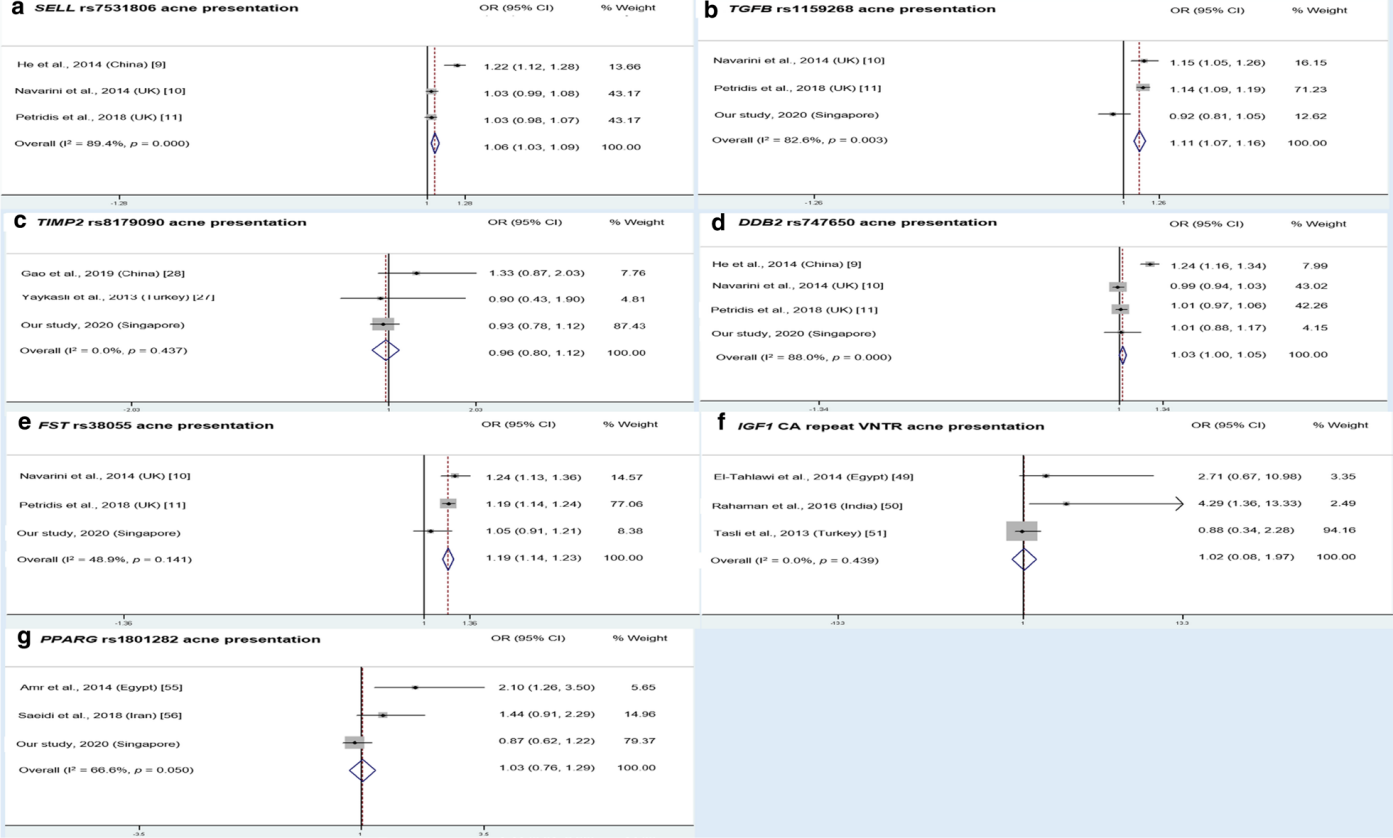
Meta-analysis of other gene variants associated with acne presentation involving immune and inflammatory responses, and sebaceous gland function and activity. **a** *SELL* rs7531806. **b** *TGFB* rs1159268. **c** *TIMP2* rs8179090. **d** *DDB2* rs747560. **e** *FST* rs38055. **f** *IGF1* CA repeat VNTR. **g** *PPARG* rs1801282. Only gene variants that were investigated in at least two other previous studies were included in the meta-analysis. Analysis was performed under allele model (minor allele *vs*. major allele), i.e. A versus G for **a**, T *vs*. A for **b**, C versus G for **c**, G versus A for **d**, A versus G for **e**, 192/192 versus non192/non192 for **f**, and G versus C for **g**. OR and CI values for **a**, **d** and **e** for Petridis et al. [11] were derived from the authors’ meta-analysis. OR and CI values for **a**, **d** for Navarini et al. [10] were derived from personal communication with the authors, while for **e** was derived from [11]

Other genes and polymorphisms that are likely to influence acne or acne severity by affecting immune and inflammatory responses are described as follows. The reviewed articles reported that *RETN* (resistin) SNPs − 420 C/G and + 299 G/A were significantly associated with the risk of acne and more severe forms of acne [29, 30], while the—− 20/ + 299 GA haplotype was significantly associated with the risk of acne [30]. Furthermore, one report found that *NLRP3* (NOD-like receptor protein 3) rs10754558, but not rs4612666, was significantly associated with acne risk [73]. Other SNPs that were significantly associated with acne presentation: *ACE* (angiotensin-converting enzyme) I/D VNTR [17], *MMP2* (Matrix metalloprotease 2) rs2285053 [28]; severe acne: *MAPK11* (mitogen-activated protein kinase 11) rs144991069, *SEMA4B* (semaphorin 4B) rs34560261, *SELL* (selectin L) rs7531806, *TGFB2* (transforming growth factor beta 2) rs1256580 [11], *TNFRSF1B* rs1061622 [32], *TRAF3IP2* (TRAF3 Interacting Protein 2) rs33980500 [26]; and teenage acne: *TYK2* (tyrosine kinase 2) rs12720356 [26]. Meta-analysis of *SELL* rs7531806 and *TGFB2* rs1256580 SNPs found that the minor alleles posed a significantly increased risk for acne—pooled OR was 1.06 (95% CI: 1.03–1.09) and 1.11 (95% CI: 1.07–1.16), respectively, albeit with significant heterogeneity observed (I^2^ = 89.4%, *p* = 0.000; I^2^ = 82.6%, *p* = 0.003, respectively; Fig. 4a, 4b).

In contrast, SNPs that showed no significant association with acne risk, include *EGFR* (epithelial growth factor receptor) variants rs2293347, rs2227983, CA repeat intron 1 VNTR [59], *ITLN1* (Intelectin-1/Omentin) Val109Asp [24], *MMP2* (matrix metallopeptidase 2) − 1306 C/T [27], *NLRP3* (NLR family pyrin domain containing 3) rs4612666 [73], and *TIMP2* (TIMP metallopeptidase inhibitor 2) − 418 G/C [27, 28]. Meta-analysis of *TIMP2* rs8179090 also confirmed no significant association with acne risk [pooled OR = 0.96; (95% CI: 0.80–1.12); no significant heterogeneity observed (I^2^ = 0%, *p* = 0.437); Fig. 4c].

### Genes and gene variants involved in sebaceous gland function and activity

Some genes implicated in acne and severe acne risk may influence the function and activity of the sebaceous gland. Two frequently studied gene families that have been implicated are the cytochrome P450 (*CYP*) gene family and the 3-beta hydroxysteroid dehydrogenase/isomerase (*HSD3B*) gene family.

#### CYP family

The *CYP* genes investigated included *CYP17* (Cytochrome P450 Family 17 Subfamily A Member 1), *CYP19A1* (Cytochrome P450 Family 19 Subfamily A Member 1) and *CYP1A1* (Cytochrome P450 Family 1 Subfamily A Member 1).

**Fig. 5:**
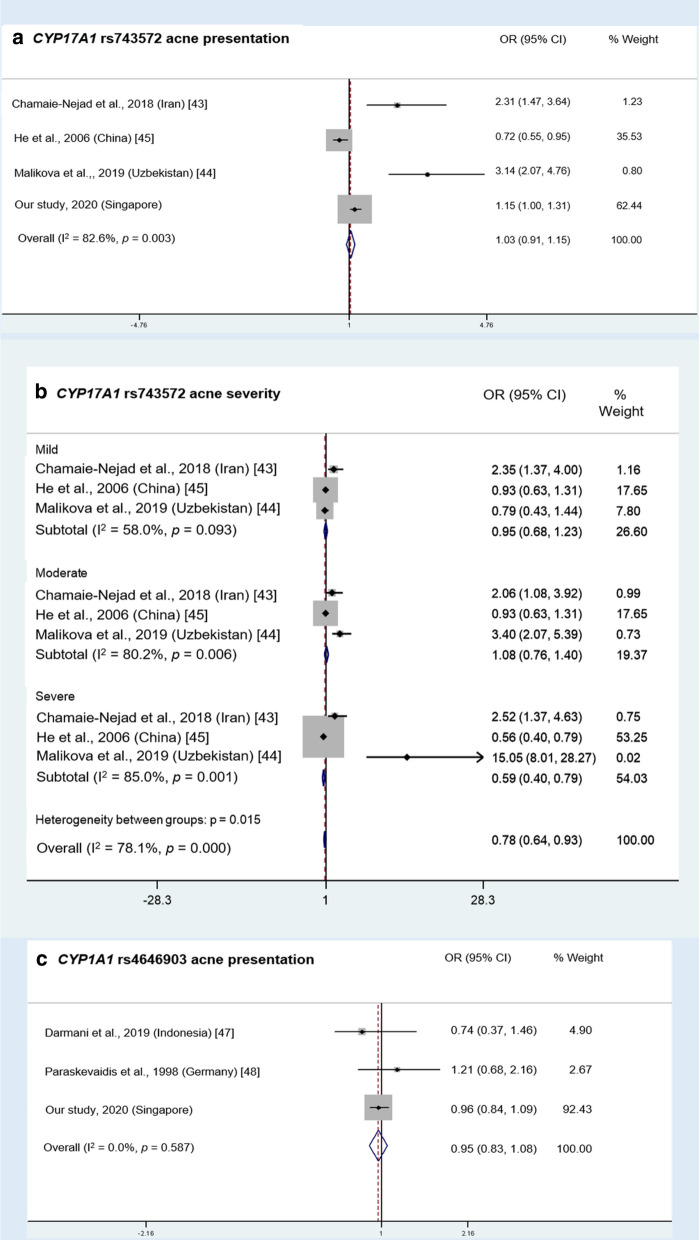
Meta-analysis of *CYP* SNPs associated with acne presentation and acne severity grades. **a** Meta-analysis of association of *CYP17A1* rs743572 with acne presentation. **b** Subgroup meta-analysis of association of rs743572 with acne severity among different grades. **c** Meta-analysis of association of *CYP1A1* rs4646903 with acne presentation. Only gene variants that were investigated in at least two other previous studies were included in the meta-analysis. Analysis was performed under allele model (minor allele *vs*. major allele)., i.e. T versus C for rs743572 and C versus T for rs464903

For *CYP17A1*, a Chinese study found that the − 34 T/C SNP was not significantly associated with both acne in the overall population and acne severity in females, but was significantly associated with acne severity in males, suggesting gender differences in acne severity genetic predisposition [45]. Another *CYP17A1* SNP rs743572 was investigated in three studies—two studies reported significant association with acne and acne severity [43, 44], while another did not [12]. Meta-analysis of these and our study for acne presentation showed that this SNP was not significantly associated with acne, as the pooled OR was 1.03 (95% CI: 0.91–1.15) (Fig. 5a), while a subgroup analysis revealed an OR of 0.95 (95% CI: 0.68–1.23), 1.08 (95% CI: 0.76–1.40), and 0.59 (95% CI: 0.40–0.79) among mild, moderate and severe grades, respectively (Fig. 5b). Significant heterogeneity was observed across these reported findings, suggesting these analyzed findings had different study outcomes across each other (all I^2^ ≥ 50%, *p* < 0.05). These heterogeneities also correspond to the publication bias detected for this SNP. Although this SNP may affect *CYP17* regulation by creating a putative novel SP1 promoter-binding site [74], in vitro EMSA experiments have so far not showed any evidence on this [75].

In addition, two *CYP19A1* SNPs, rs2236722 and rs700518, were significantly associated with acne presentation and severity [43, 46]. Two *CYP1A1* candidate SNPs were also studied where no significant association with acne was found: + 4889 A/G SNP in the German population [48] and + 6235 T/C SNP (rs4646903) in Indonesian [47], German [48] populations and in our meta-analysis [pooled OR: 0.95, 95% CI: 0.83, 1.08; no significant heterogeneity observed (I^2^ = 0%, *p* = 0.587); Fig. 5c].

#### HSD3B family

Three members of the 3-β HSD family were investigated in the reviewed studies. A Chinese study found that *HSD3B1* (Hydroxy-Delta-5-Steroid Dehydrogenase, 3 Beta- and Steroid Delta-Isomerase 1) rs6428829 and AAT haplotype, and *HSD17B3* (Hydroxysteroid 17-Beta Dehydrogenase 3) H8 haplotype were significantly associated with acne risk [53]. In contrast, a study in Egypt found that *HSD11B1* rs12086634 variants were associated with acne risk while the relationship between *HSD11B1* rs846910 variants and acne risk was unclear [52].

#### Other genes

Other frequently studied genes thought to influence sebaceous gland function and activity include the androgen receptor (*AR*), peroxisome proliferator activated receptor gamma (*PPARG)* and insulin like growth factor 1 (*IGF-1*) genes. For *AR*, a Chinese study observed that GGN (Gly) repeat VNTR was not significantly associated with acne risk [39]. However, for the CAG (Gln) repeat VNTR, a significant association with acne risk was observed in one study [39] while other studies showed that it was significantly associated with acne and acne severity in males, but not in females [40] and vice versa [41]. This suggests that there may be gender differences in the association between *AR* CAG repeat VNTR and both acne and acne severity. Biologically, these VNTRs cause variation in the lengths of polyGln and polyGly stretch in the N-terminal domain of AR receptor. The variations in repeat lengths in both VNTRs have been shown to be associated with subtle modulations of *AR* expression, resulting in the modified transcriptional activity of various downstream targets. The transcriptional activity of *AR* is inversely correlated with number of the polyGln repeat length; shorter alleles exhibit greatest activity [76–78]. Furthermore, in vitro results suggest that acne patients carrying fewer CAG repeats may exhibit a higher AR mRNA and protein expression, leading to higher sensitivity to androgens than in control individuals [79].

For *PPARG*, a study in Iran found that rs3856806 and rs1801282 SNPs were not associated with acne and acne severity in the overall population [56]. However, the study found that rs1801282 was associated with acne risk in a subset of patients who developed acne at the age of 20 or older. Another study observed that the Pro/Ala genotype of rs1801282 appeared to be protective for acne and more severe acne [55]. However, our meta-analysis revealed that the rs1801282 G allele do not pose any increased/decreased risk for acne [pooled OR: 1.03, 95% CI: 0.76, 1.29; significant heterogeneity observed (I^2^ = 66.6%, *p* = 0.050); Fig. 4g]. This heterogeneity also corresponds to the publication bias detected for this SNP. Similarly, with reference to *IGF1*, the length of CA repeats was found to be significantly associated with acne risk only [49], and the risk of both acne and more severe forms of acne [50, 51]. However, our meta-analysis revealed that the *IGF1* VNTR 192/192 genotype do not pose any increased/decreased risk for acne [pooled OR: 1.02, 95% CI: 0.08, 1.97; no significant heterogeneity observed (I^2^ = 0%, *p* = 0.439); Fig. 4f].

Gene variants that were significantly associated with severe acne risk include: *DDB2* (damage specific DNA binding protein 2) rs747650 and rs1060573 [10], *MUC1* (mucin 1) 20aa VNTR [54], *FST* (follistatin) rs629725, *LAMC2* (laminin subunit gamma 2) rs10911268, *LGR6* (leucine rich repeat containing G protein-coupled receptor 6) rs788790, *OVOL1* (ovo like transcriptional repressor 1) rs144991069, *SPECC1L* (sperm antigen with calponin homology and coiled-coil domains 1 like) rs28360612, and *WNT10A* (Wnt family member 10A) rs121908120 [11]. Our meta-analysis revealed that the *FST* rs629725 A allele poses a significantly modest increased risk for acne [pooled OR: 1.19, 95% CI: 1.14, 1.23; no significant heterogeneity observed (I^2^ = 48.9%, *p* = 0.141); Fig. 4e], while the *DDB2* rs747650 G allele also poses a negligible increased risk for acne [pooled OR: 1.03 (95% CI: 1.00, 1.05), albeit with significant heterogeneity observed (I^2^ = 88.0%, *p* = 0.000); Fig. 4d].

In addition, the following variants were significantly associated with acne risk: *ADH7* (alcohol dehydrogenase 7) rs1154469 [38], *SRD5A2* (steroid 5 alpha-reductase 2) TA repeat VNTR [57] and *VDR* (vitamin D receptor) rs731236 and rs7975232 [58]. However, no significant association emerged between *SRD5A2* V89L SNP and acne risk [57]. All these genes have been implicated in sebaceous gland function and activity.

## Discussion

**Fig. 6:**
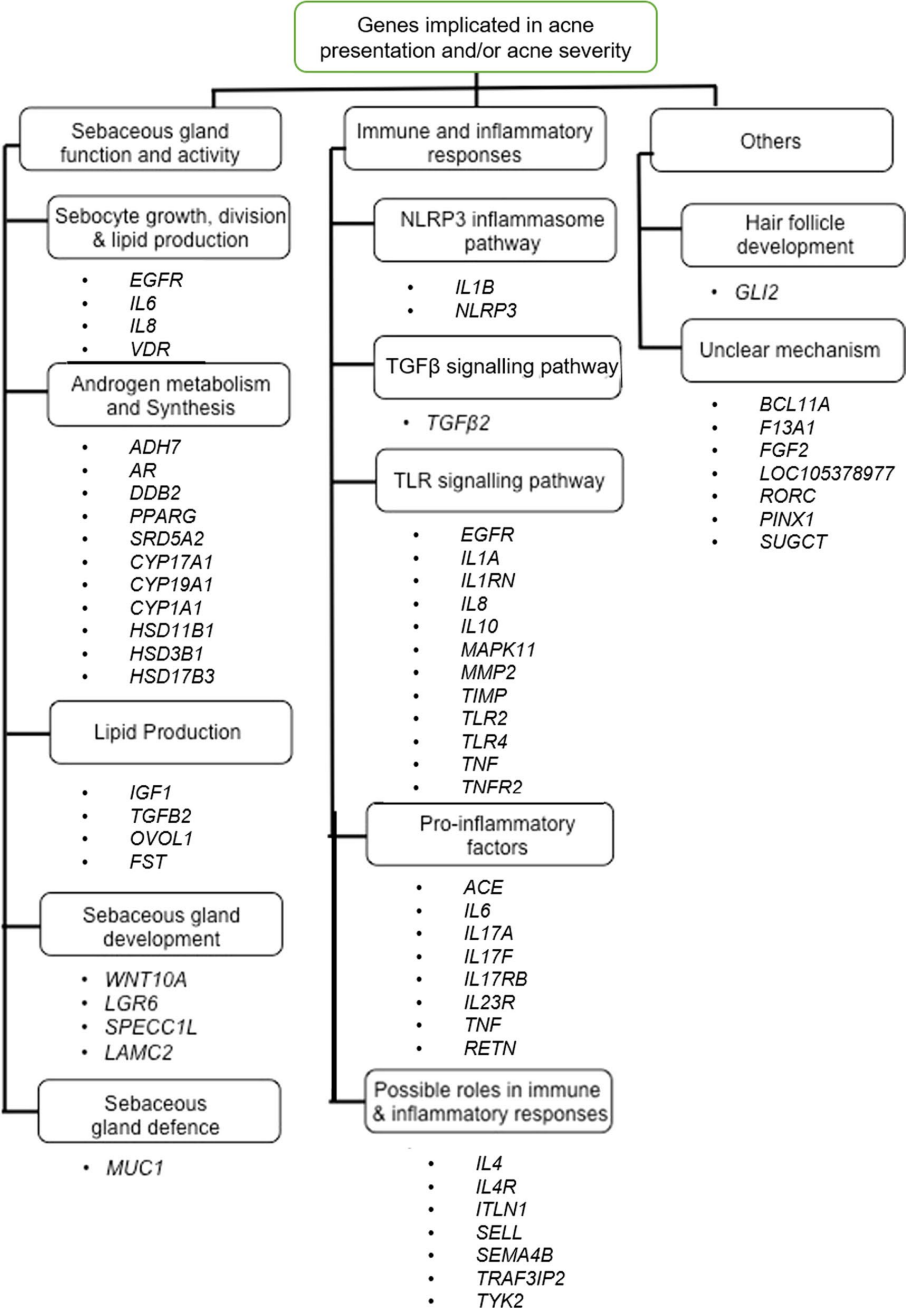
Flowchart depicting potential mechanisms by which genes implicated in the reviewed studies may influence the risk of acne presentation or acne severity

Figure 6 summarizes the acne-implicated genes and their potential biological functions in the pathogenesis of acne. These biological functions are also part of the top DAVID functional annotation clusters, as analyzed earlier. Notably, some of these genes have overlapping functions. The following parts will further discuss how these acne-implicated genes play plausible roles in the biological pathways influencing inflammation, sebaceous gland function and activity.

### Immune and inflammatory responses

A contributing factor to the pathogenesis of acne is *C. acnes*, a species of bacteria commonly found in pilosebaceous units that is thought to promote the development of acne lesions [80]. Studies suggest that *C. acnes* can activate the NLRP3 inflammasome pathway, eventually resulting in elevated IL-1β levels and contributing to the pathogenesis of acne [81]. In addition, TGFβ may not only have roles in sebocyte lipid synthesis, but may also regulate immune responses against *C. acnes*. Furthermore, *C. acnes* can be recognized by pattern recognition receptors such as TLR2 and TLR4, which mount immune responses against the bacteria [80], thus influencing acne development. Upon recognizing *C. acnes*, TLRs can activate cellular components such as TNF and IL-1A, which in turn activate chemokines such as IL-8, IL-10 and downstream components that eventually promote inflammation [13, 24]. IL-1A activity may also be modulated by IL1RN [14].

Furthermore, TNF can interact with its receptors, such as TNFR2, activating intracellular signaling cascades. These cascades can activate MAPKs such as MAPK11 that upregulate the expression of pro-inflammatory mediators, and also activate matrix MMPs such as MMP-2 to bring about tissue remodeling [82]. MMP activity is regulated by TIMPs; altering the balance of MMP and TIMP activity may contribute to acne development [27]. Similarly, IL-6 (which could be stimulated by IL17A, IL17F and ACE) is reported play a role in modulating host defense mechanisms [14] and inflammation [83].

Pro-inflammatory factors TNF and IL-6 also have roles in modulating *RETN* transcription and translation. Studies demonstrate a bidirectional relationship between resistin and TNF and IL-6: TNF and IL-6 not only modulate resistin expression but their activity is in turn modulated by resistin levels [83]. Variation in any of these genes can affect their expression, function or activity, which may predispose individuals to acne or severe acne.

EGF/EGFR signaling plays an important role in the regulation of inflammation by inhibiting proinflammatory cytokines including IL-1α, IL-8 and TNF-α in *C. acnes* stimulated epidermal keratinocytes [84].

While the specific roles of IL-4 and IL-4R in the pathogenesis of acne are unclear, these proteins are known to play a role in inflammatory responses. Thus it is hypothesized that IL-4 and IL-4R are involved in the inflammatory processes associated with acne [23]. In addition, studies have shown that adipokines secreted by adipocytes may play a role in inflammation associated with acne [25]. The adipokine omentin may thus influence inflammatory processes that contribute to the pathogenesis of acne, such as by preventing inflammation caused by TNF [25]. Similarly, L-selectin is expressed in certain classes of immune cells and plays a role in immune cell invasion of inflamed tissue [9]. Evidence suggests that *SELL*, which encodes L-selectin, may contribute to inflammatory processes associated with more severe acne [14]. In addition, while the role of SEMA4B in acne pathogenesis is uncertain, it is known to be involved in immune responses and downregulates the secretion of IL-4 and IL-6 in basophils [85]. Thus, it is possible that SEMA4B may also play a role in acne-related immune processes.

Finally, two recent bioinformatics studies using gene expression data of the lesional and nonlesional skin of acne patients from the NCBI Gene Expression Omnibus found 12 top upregulated differentially expressed genes (DEGs) involved in immune and inflammatory responses, namely *FPR2*, *ITGB2*, *CXCL8*, *C3AR1*, *CXCL1*, *FCER1G*, *LILRB2*, *PTPRC*, *SAA1*, *CCR2*, *ICAM1*, and *FPR1* [86, 87]. Specifically, the putative pathways involved in acne pathogenesis include chemokine signaling, cytokine–cytokine receptor interaction, and Fc gamma R-mediated phagocytosis [86, 87]. Of note, neither of the variants in these genes have been investigated in candidate gene studies nor were picked up by GWAS for association with acne presentation and severity, thus warrants future investigation.

### Sebaceous gland function and activity

Sebaceous glands, which are involved in the biosynthesis and secretion of sebum, are present on almost the entire body surface, and particularly on the face [88]. Changes in the activity of these glands may lead to changes in skin physiology and have been implicated in the pathogenesis of acne. Increased sebaceous gland activity can result in higher sebum levels, which may hamper normal cellular processes in the skin and eventually lead to the development of acne lesions [88].

Some of the genes found to be associated with acne and acne severity in this review may have roles in the sebaceous gland function and activity, and thus variations in these genes may affect cell signaling and functioning in sebaceous glands, thus contributing to acne development or exacerbating acne. One cell-signaling pathway in sebaceous gland cells (sebocytes) is the VDR pathway. Sebocytes have been reported to express VDR, and studies have shown that VDR activation affects sebocyte growth and division, lipid production and levels of ILs, such as IL-6 and IL-8 [89]. Thus, variants in *VDR*, *IL6* and *IL8* may affect sebaceous gland function and contribute to acne development or exacerbation.

Two other pathways, the AR and PPAR signaling pathways, may also regulate sebocyte function and activity. Sebocytes have important functions in androgen metabolism, and high androgen levels can upregulate the activity of sebocytes. The effects of androgens on the sebaceous gland are thought to be mediated via AR and PPAR signaling pathways [88]. AR activity may, in turn, be regulated by *DDB2*. The protein encoded by *DDB2* facilitates interactions between AR and a complex responsible for targeting AR for ubiquitination and degradation [14]. In addition, variations in genes involved in androgen synthesis can affect androgen levels and thus influence sebocyte activity. The gene *SRD5A2* [57], some members of the *CYP* gene family, such as *CYP17A1* [13], and some members of the *HSD* gene family encode enzymes that have roles in androgen synthesis [53]. For example, the enzymes encoded by *HSD3B1* and *HSD17B3* are involved in the biosynthesis of testosterone [53].

In addition, variations in genes such as *IGF1* and *TGFB* are suggested to influence the production of lipids in sebocytes, and may contribute to acne development [14]. Notably, dietary variables such as intake of fish, fruits and vegetables were suggested to influence acne and severe acne risk by modulating IGF-1 levels, showing a possible interaction between genetic factors and modifiable risk factors [90]. In addition, the activity of the TGFβ signaling pathway is regulated by the transcription factor *OVOL1* and the protein follistatin (encoded by *FST*), thus variations in these genes may influence sebocyte lipid synthesis and thus play a part in acne development and exacerbation [14]. Navarini et al. [10] also revealed that transcript levels of *TGFB2* and *OVOL1* were significantly decreased in fresh inflammatory acne papules compared to normal skin. *MUC1* is another gene expressed in sebaceous glands and it encodes for a glycoprotein that may be involved in inhibiting the adhesion of bacteria onto the skin, including the acne-causing bacteria *C. acnes* [54]. Variations in *MUC1* may influence the anti-bacterial properties of the glycoprotein, thus influencing acne development.

Furthermore, reports have suggested that some genes implicated in the pathogenesis of acne may be involved in the development of sebaceous glands [91]. One of these genes is *WNT10A*. *WNT10A* activity can influence sebum production in the sebaceous gland [11] and may also regulate the differentiation of progenitors to form sebocytes [91]. Thus, changes in Wnt signaling may affect the normal development and functioning of the sebaceous gland and in turn, the risk of acne or severe acne. Wnt signaling is regulated by *LGR6*, and high *LGR6* expression can inhibit sebaceous progenitor differentiation [91]. *SPECC1L* is also reported to be a component of the Wnt signaling pathway [92] and may be implicated in acne development. Another gene involved in the development of sebaceous glands is *LAMC2*, which facilitates the migration of sebaceous gland progenitor cells to the site of the gland [91]. Variations in these genes can affect sebaceous gland development, contributing to acne development and exacerbation.

Recently, *ADH7* rs1154469 has been identified as a novel susceptibility locus for severe acne among Han Chinese [38]. The most active enzyme encoded by this gene is retinol dehydrogenase; thus it may participate in the synthesis of retinoic acid. Retinoic acid is a retinoid which acts to normalize desquamation within the sebaceous follicles that leads to obstruction of the pilosebaceous canal as seen in acne, by reducing keratinocyte proliferation and promoting differentiation [93].

Notably, environments with high sebum levels favor the growth of *C. acnes* and promote the colonization of pilosebaceous units by *C. acnes* [94]. This suggests a link between the two implicated pathways: changes in the sebaceous gland that lead to increased sebum production may promote *C. acnes* infection, triggering inflammatory and immune responses that contribute to acne pathogenesis.

### Other genes and potential pathways implicated in acne

The rest of the genes identified in the reviewed articles were not implicated in inflammation, immune responses or sebaceous gland function and activity. The genes and SNPs significantly associated with severe acne found by Petridis et al. [11] were: *BCL11A* (BAF chromatin remodeling complex subunit BCL11A) rs2901000, *FGF2* (fibroblast growth factor 2) rs4487353, *GLI2* (GLI family zinc finger 2) rs1092479, LOC105378977 rs158639, *PINX1* (PIN2 (TERF1) interacting telomerase inhibitor 1) rs28570522 and *SUGCT* (succinyl-CoA:glutarate-CoA transferase) rs7809981; and by Yang et al. [38]: *F13A1* (Coagulation Factor XIII A Chain) rs435048. Furthermore, a few SNPs that were not located within a gene were investigated in the reviewed studies. The SNPs rs2727365, rs4133274 and rs13248513 SNPs showed a significant association with severe acne risk [11, 12]; the mechanism(s) by which variations at these polymorphisms influence severe acne risk are unclear.

It is also unclear how *PINX1*, which has functions in telomere length maintenance and chromosome stability [95]; *F13A1,* which encodes coagulation factor XIII, the last zymogen to become activated in the blood coagulation cascade and a transglutaminase enzyme [96]; *BCL11A*, a transcription factor involved in hematopoietic development [97]; *FGF2*, which is involved in processes that contribute to scar formation and wound healing [11]; and *SUGCT*, which encodes an enzyme that converts glutarate to glutaryl-CoA [98] may influence the pathogenesis of acne.

Genes implicated in acne presentation and severity may influence the pathogenesis of acne through mechanisms other than altering sebaceous gland function or inflammatory and immune responses. For instance, the transcription factor *GLI2* regulates the expression of sonic hedgehog (Shh), a component of signaling pathways that influence the development of hair follicles [99]. A previous study reported that several genes involved in hair follicle development were implicated in severe acne risk, suggesting that this pathway may contribute to the pathogenesis of acne [11].

## Conclusions

The reviewed papers reported on genes and their variants associated with the risk of acne presentation and severity. However, the results of different studies show inconsistencies, with some studies reporting that a particular variant increases the risk, while others did not. Out of the 51 studies reviewed, only five (including ours) were GWAS while others where candidate gene studies. Hence, we acknowledge the poor reproducibility of association studies, which could be attributed to factors such as insufficient availability of genetic markers, inadequate handling of population structure, lack of statistical power due to low sample size, improper control of multiple testing and extensive publication bias [reviewed in [100]]. Furthermore, it is also possible that rather than particular SNPs, it is the haplotype of multiple SNPs or changes in gene expression or function that is associated with acne presentation or severity. Rather than considering the impact of individual variants on acne presentation or severity, it may be more helpful to consider the gene as a functional unit and to understand the effects of altered gene function on acne presentation and severity.

In conclusion, this review summarizes the literature on candidate genes implicated in the risk of acne presentation and severity and possible mechanisms by which these genes may affect acne pathogenesis. Notably, a large majority of the candidate genes identified are suggested to have roles in the function and activity of sebaceous glands or immune and inflammatory responses—in line with the literature that describes acne as a chronic inflammatory disease of the pilosebaceous unit. Understanding the genetic susceptibility factors and biological pathways involved in the pathogenesis of acne will help us to gain insights into developing effective acne treatments.

## Supplementary Information

**Additional file 1: Table S1**. PRISMA Checklists.

**Additional file 2: Table S2.1**. Comparison of the genes associated with acne in the genetics review and the genes associated with acne using data extracted from our existing Singapore GWAS database; **Table S2.2**. SNPs in *TLR4* that were significantly associated with acne using data extracted from our existing Singapore GWAS database. **Figure S1**. Positions of the SNPs significantly associated with acne using data extracted from our existing Singapore GWAS database with reference to the location of *TLR4*.

**Additional file 3: Figure S2**. Gene ontology analysis/network analysis was performed using the online Database for Annotation, Visualization and Integrated Discovery (DAVID) v6.8 software; **Figure S3**. Assessment of publication bias using Begg’s funnel plots and Egger’s test for the SNPs included in the meta-analysis.

## Data Availability

All data used and included in this study are available from the corresponding author (F.T.C.).

## Abbreviations

ACE: Angiotensin-converting enzyme
ADH7: Alcohol dehydrogenase 7
AR: Androgen receptor
BCL11A: BAF chromatin remodeling complex subunit BCL11A
CI: Confidence intervals
CYP17: Cytochrome P450 family 17 subfamily A member 1
CYP19A1: Cytochrome P450 family 19 subfamily A member 1
CYP1A1: Cytochrome P450 family 1 subfamily A member 1
DDB2: Damage specific DNA binding protein 2
EGFR: Epithelial growth factor receptor
F13A1: Coagulation factor XIII A chain
FGF2: Fibroblast growth factor 2
FST: Follistatin
GLI2: GLI family zinc finger 2
GWAS: Genome-wide association study
HSD17B3: Hydroxysteroid 17-beta dehydrogenase
3HSD3B1: Hydroxy-delta-5-steroid dehydrogenase, 3 beta- and steroid delta-isomerase
HWE: Hardy–Weinberg equilibrium
IGF-1: Insulin like growth factor 1
IL10: Interleukin-10
IL17A: Interleukin-17A
IL17F: Interleukin-17F
IL17RB: Interleukin-17 receptor B
IL1A: Interleukin-1α
IL1B: Interleukin-1β
IL1RN: Interleukin 1 receptor antagonist
IL23R: Interleukin-23 receptor
IL4: Interleukin-4
IL4R: Interleukin-4 receptor
IL6: Interleukin-6
IL8: Interleukin-8
ITLN1: Intelectin-1/omentin)
LAMC2: Laminin subunit gamma 2
LGR6: Leucine rich repeat containing G protein-coupled receptor 6
MAPK: Mitogen-activated protein kinase
MAPK11: Mitogen-activated protein kinase 11
MMP-2: Matrix metallopeptidase 2
MMP2: Matrix metalloprotease 2
MUC1: Mucin 1
NLRP3: NLR family pyrin domain containing 3
NLRP3: NOD-like receptor protein 3
OR: Odds ratio
OVOL1: Ovo like transcriptional repressor 1
PINX1: PIN2 (TERF1) interacting telomerase inhibitor 1
PPARG: Peroxisome proliferator activated receptor gamma
RETN: Resistin
SELL: Selectin L
SEMA4B: Semaphorin 4B
SNP: Single nucleotide polymorphism
SPECC1L: Sperm antigen with calponin homology and coiled-coil domains 1 like
SRD5A2: Steroid 5 alpha-reductase 2
SUGCT: Succinyl-CoA:glutarate-CoA transferase
TGFB2: Transforming growth factor beta 2
TIMP2: TIMP metallopeptidase inhibitor 2
TLR2: Toll-like receptor 2
TLR4: Toll-like receptor 4
TNF: Tumor necrosis factor
TNFR2: Tumor necrosis factor receptor 2
TRAF3IP2: TRAF3 interacting protein 2
TYK2: Tyrosine kinase 2
VDR: Vitamin D receptor
WNT10A: Wnt family member 10A
