# Supplementary material for: Gene variants associated with acne vulgaris presentation and severity: a systematic review and meta-analysis
Source: BMC Med Genomics. 2021 Apr 13;14:103. doi: 10.1186/s12920-021-00953-8 (PMC8045239; doi:10.1186/s12920-021-00953-8)
Supplement: Supplementary file 2 — Additional file 2: Table S2.1. Comparison of the genes associated with acne in the genetics review and the genes associated with acne using data extracted from our existing Singapore GWAS database; Table S2.2. SNPs in TLR4 that were significantly associated with acne using data extracted from our existing Singapore GWAS database. Figure S1. Positions of the SNPs significantly associated with acne using data extracted from our existing Singapore GWAS database with reference to the location of TLR4. [file 12920_2021_953_MOESM2_ESM.docx]

**Title: Gene variants associated with acne vulgaris presentation and severity: a systematic review and meta-analysis**

Anna Hwee Sing Heng, Yee-How Say, Yang Yie Sio, Yu Ting Ng, Fook Tim Chew

**Additional File 2**

**Subset of genotyping data of acne cases and controls extracted from our existing GWAS database on allergic and skin disease among Singapore Chinese**

**Methods**

***Participants and data collection***

The sample used for the genome-wide association study (GWAS) was obtained from a cross-sectional study conducted in Singapore in 2014, 2015 and 2019. The sample was made up of volunteers who were staff and students of the National University of Singapore. To avoid sampling bias, non-Singapore Chinese were excluded. Mouthwash samples were collected from the volunteers and the DNA extracted from the mouthwash samples were used for GWAS. Responses to two questions in an epidemiology questionnaire were used to classify participants as acne cases and controls. The two questions were (1) “Have you ever visited a doctor for your acne condition?’ and (2) “Do you have scars (keloids) left by acne/boils?” Participants who responded ‘Yes’ to either question were classified as acne cases while participants who responded ‘No’ to both questions were classified as acne controls. Genotyping data were extracted from 982 acne cases and 846 controls.

Ethical approval for participant recruitment in Singapore was granted from the Institutional Review Board of NUS (NUS-IRB Ref-Code: B-10-343 and B-14-150 for genomic DNA and blood collection, respectively; NUS-IRB Ref-Code: 07–023, 09–256, 10–445, and 13–075 for the large scale epidemiology and genetics study) and the National Healthcare Group Domain Specific Review Board (B/04/055). All participants (or parent/guardian for those below 21 years) signed informed consent forms, and the study was conducted in accordance with the Declaration of Helsinki.

***SNP-array genotyping***

Genomic DNA was extracted from either PBMCs or mouthwash samples using the Axygen^®^ AxyPrep™ Multisource Genomic Miniprep DNA kit (Axygen, CA). SNP-array genotyping was performed using the Infinium OmniZhongHua-8 v1.3 BeadChip platform. Haplotype phasing and imputations of the data were performed using the IMPUTE2 program. Genotyping was performed by the University of Utah Genomics Core Facility (Salt Lake City, Utah) using the BeadXpress system (Illumina, Inc, San Diego, CA) and data were analyzed using the GenomeStudio software.

***Statistical analysis***

Single nucleotide polymorphisms (SNPs) with a *p-*value > 0.001 in the Hardy-Weinberg test of equilibrium and minor allele frequency (MAF) > 0.05 were imputed. Further quality control of SNPs was conducted, and SNPs that fulfilled any of the following criteria were excluded: (1) SNPs with a call rate below 90%; (2) monomorphic SNPs; (3) duplicated SNPs; (4) Insertions and Deletions. In addition, since this GWAS was conducted as a comparison dataset for cross-comparison with the genetics review (Section 5), only SNPs located within 2 kilobases of the any of the candidate genes investigated in the genetics review were investigated in this GWAS. After exclusion, a total of 4517 SNPs were analysed.

Logistic regression was conducted using the plink software to investigate the association between the minor allele counts and acne presentation. The raw *p*-values obtained from logistic regression analyses were adjusted using the Benjamini-Hochberg correction for false discovery rate in R Version 3.3.3. In addition, the UCSC (University of California Santa Cruz) Genome Browser was used to generate Supplementary Figure S1.

**Results**

The genes investigated in the genetics review were also investigated using data extracted from our existing Singapore GWAS database. Supplementary Table S2.1 describes the association of these genes with acne presentation using data extracted from our existing Singapore GWAS database. Only variants in several single nucleotide polymorphisms (SNPS) located within 2 kilobases of the *TLR4* gene demonstrated significant association with acne presentation (*p=*0.021222 for all investigated SNPs). A full list of the SNPs located near *TLR4* that showed significant association with acne presentation can be found in Supplementary Table S2.2. Supplementary Figure S1 is a diagrammatic representation of the *TLR4* gene and the locations of the SNPs significantly associated with acne presentation. Notably, all the SNPs are located within the 3’ untranslated region of the *TLR4* gene. In contrast, genetic variants in the other investigated genes showed no significant association with acne presentation using data extracted from our existing Singapore GWAS database.

**Supplementary Table S2.1** Comparison of the genes associated with acne in the genetics review and the genes associated with acne using data extracted from our existing Singapore GWAS database.

| Genes* investigated in the review | |
| --- | --- |
| Several SNPs in Singapore GWAS significantly associated with acne | SNPs significantly associated with acne in other studies but not in Singapore GWAS |
| *TLR4* | *BCL11A; CXCL8; CYP1A1; CYP17; CYP19; DDB2; FGF2; FST; GLI2; HSD11B1; HSD17B3; HSD3B1; IGF1; IL10; IL1A; IL1B; IL1RN; IL4; IL4R; IL6; ITLN1; LAMC2; LAMC2; LGR6; LOC105378977; MMP2; MUC1; NLRP3; OVOL1; PINX1; PPARG; RETN; SELL; SEMA4B; SPECC1L; SRD5A2; SUGCT; TGFB2; TIMP2; TLR2; TNF; TNFRSF1B; VDR; WNT10A* |

*Only genes investigated in both the review and the Singapore GWAS are presented in this table; not all reviewed genes are represented.

Abbreviations: GWAS (genome-wide association study); SNP (single nucleotide polymorphism)

**Supplementary Table S2.2** SNPs in *TLR4* that were significantly associated with acne using data extracted from our existing Singapore GWAS database.

| **SNP** | **OR** | **95% CI** | ***p-*value^a^** | **eQTL availability from GTex Portal; tissue type** |
| --- | --- | --- | --- | --- |
| rs1554973 | 1.324 | 1.111-1.577 | 0.021222 | Yes; Esophagus - Muscularis |
| rs752998 | 1.305 | 1.095-1.556 | 0.021222 | Yes; Esophagus - Muscularis |
| kgp7425198 | 1.325 | 1.077-1.63 | 0.021222 | No |
| rs72616621 | 1.318 | 1.073-1.62 | 0.021222 | No |
| rs11536891 | 1.31 | 1.067-1.608 | 0.021222 | Yes; Esophagus – Muscularis, Whole Blood, Artery - Tibial |
| rs2183016 | 1.31 | 1.067-1.609 | 0.021222 | Yes; Esophagus – Muscularis, Whole Blood, Artery - Tibial |
| rs7045953 | 1.31 | 1.067-1.609 | 0.021222 | Yes; Esophagus – Muscularis, Whole Blood, Artery - Tibial |
| rs7873784 | 1.307 | 1.064-1.605 | 0.021222 | Yes; Esophagus – Muscularis, Whole Blood, Artery - Tibial |
| rs17420985 | 1.305 | 1.063-1.603 | 0.021222 | Yes; Esophagus – Muscularis, Whole Blood, Artery - Tibial |
| rs60103585 | 1.305 | 1.063-1.603 | 0.021222 | Yes; Esophagus – Muscularis, Whole Blood, Artery – Tibial, Heart - Atrial Appendage |
| rs11536896 | 1.305 | 1.063-1.603 | 0.021222 | Yes; Esophagus – Muscularis, Whole Blood, Artery - Tibial |
| rs72616620 | 1.305 | 1.063-1.604 | 0.021222 | No |
| rs7044464 | 1.303 | 1.061-1.601 | 0.021222 | Yes; Esophagus – Muscularis, Whole Blood, Artery - Tibial |
| rs7856729 | 1.302 | 1.06-1.599 | 0.021222 | Yes; Esophagus – Muscularis, Whole Blood, Artery - Tibial |
| rs11536898 | 1.302 | 1.06-1.599 | 0.021222 | Yes; Esophagus – Muscularis, Whole Blood, Artery – Tibial, Heart - Atrial Appendage |

Abbreviations: GWAS (genome-wide association study); SNP (single nucleotide polymorphism); OR (odds ratio); CI (confidence interval); eQTL (expression quantitative trait loci)

^a^*p*-values adjusted for false discovery rate using the Benjamini-Hochberg correction


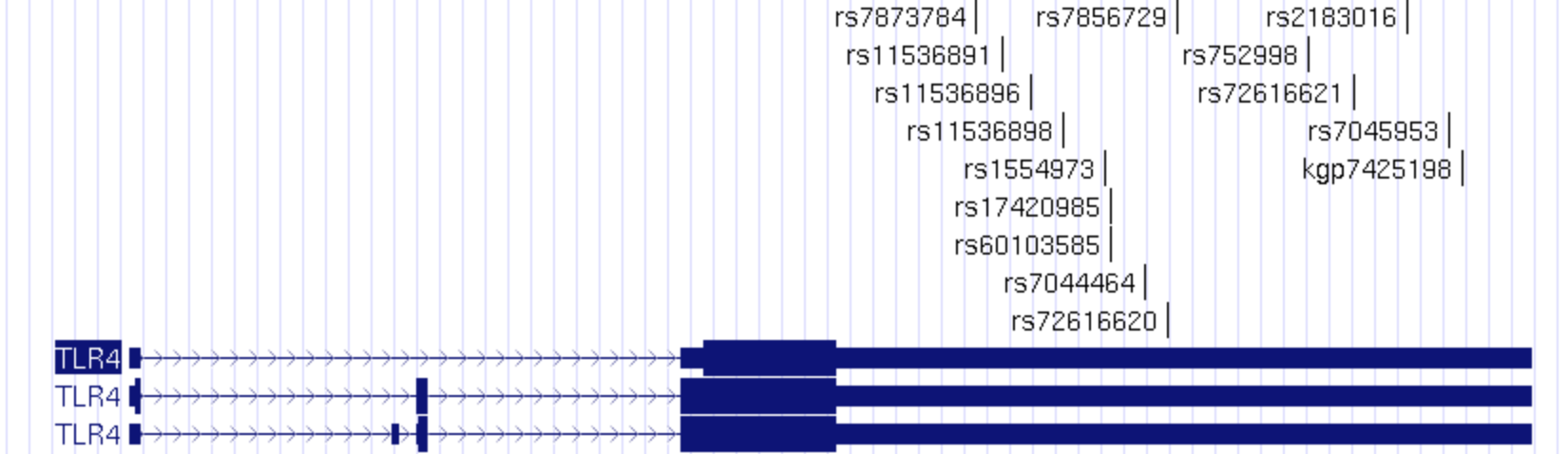


5’

3’


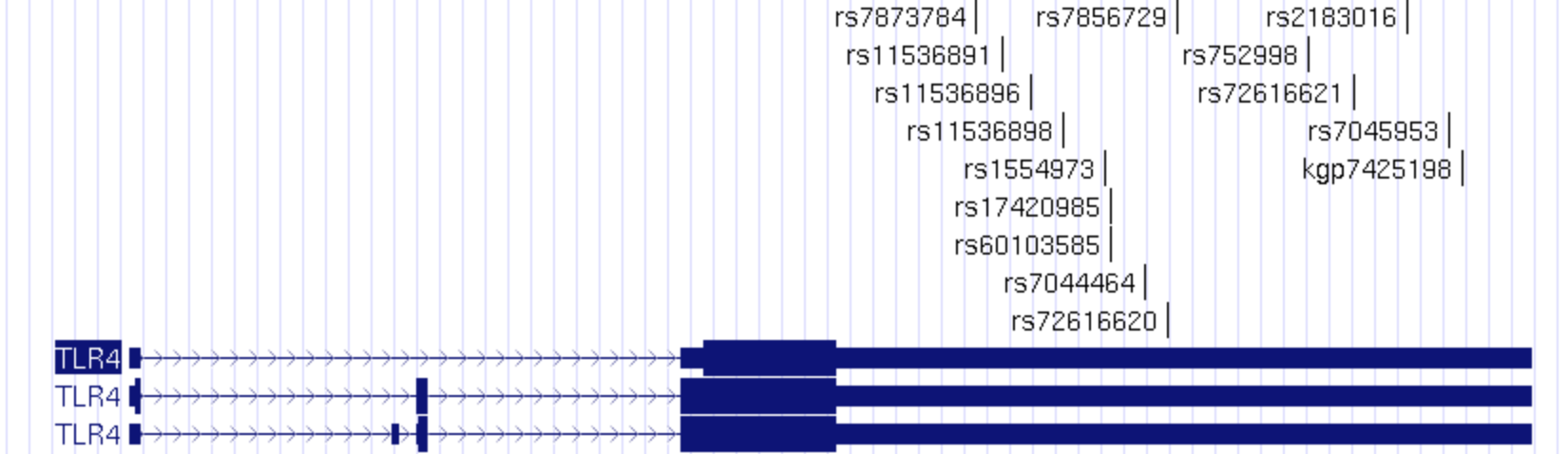

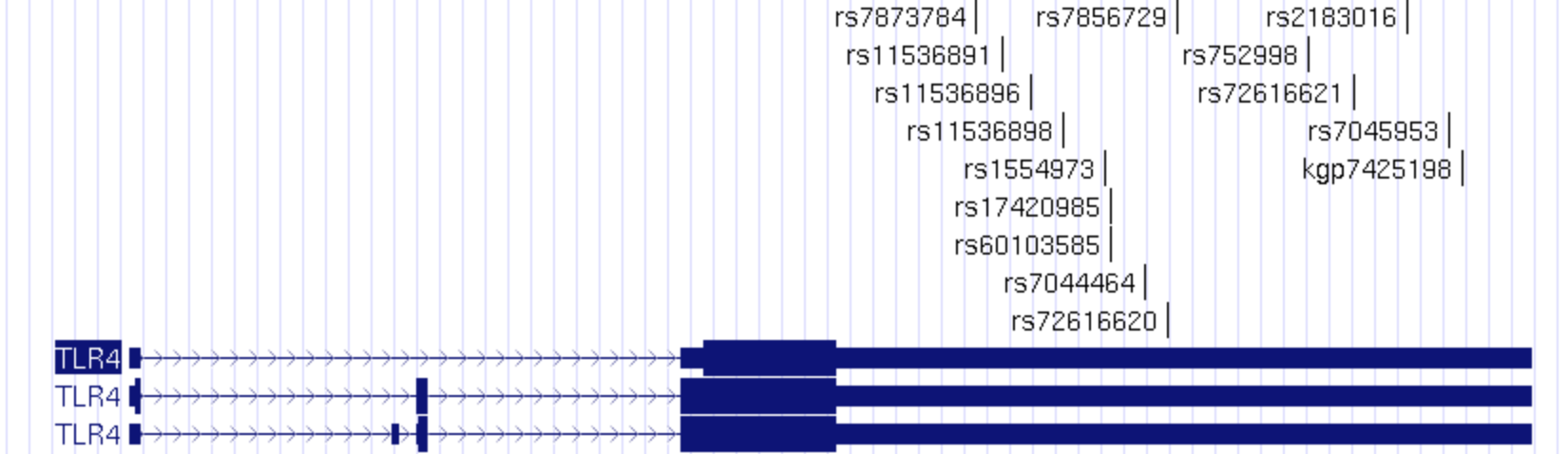

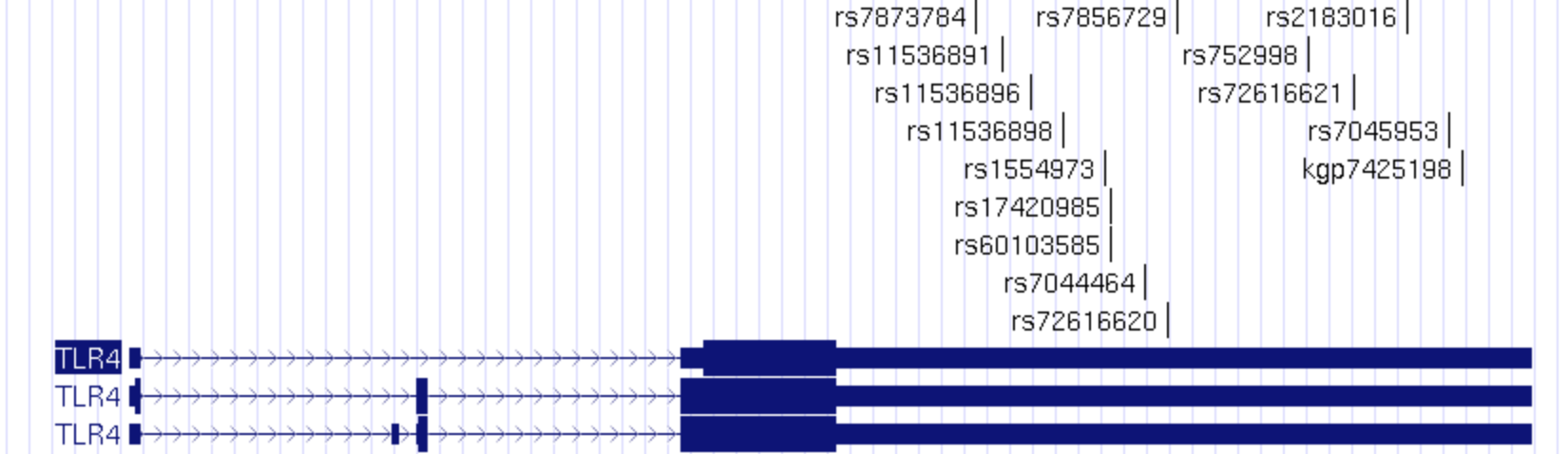


Legend:

Thin dashes or bars: Untranslated region (UTR)

Thick dashes or bars: Exons

Lines with arrows: Introns

**Supplementary Figure S1** Positions of the SNPs significantly associated with acne using data extracted from our existing Singapore GWAS database with reference to the location of *TLR4.*
