# Supplementary material for: Gene variants associated with acne vulgaris presentation and severity: a systematic review and meta-analysis
Source: BMC Med Genomics. 2021 Apr 13;14:103. doi: 10.1186/s12920-021-00953-8 (PMC8045239; doi:10.1186/s12920-021-00953-8)
Supplement: Supplementary file 3 — Additional file 3: Figure S2. Gene ontology analysis/network analysis was performed using the online Database for Annotation, Visualization and Integrated Discovery (DAVID) v6.8 software; Figure S3. Assessment of publication bias using Begg’s funnel plots and Egger’s test for the SNPs included in the meta-analysis. [file 12920_2021_953_MOESM3_ESM.docx]

**Title: Gene variants associated with acne vulgaris presentation and severity: a systematic review and meta-analysis**

Anna Hwee Sing Heng, Yee-How Say, Yang Yie Sio, Yu Ting Ng, Fook Tim Chew


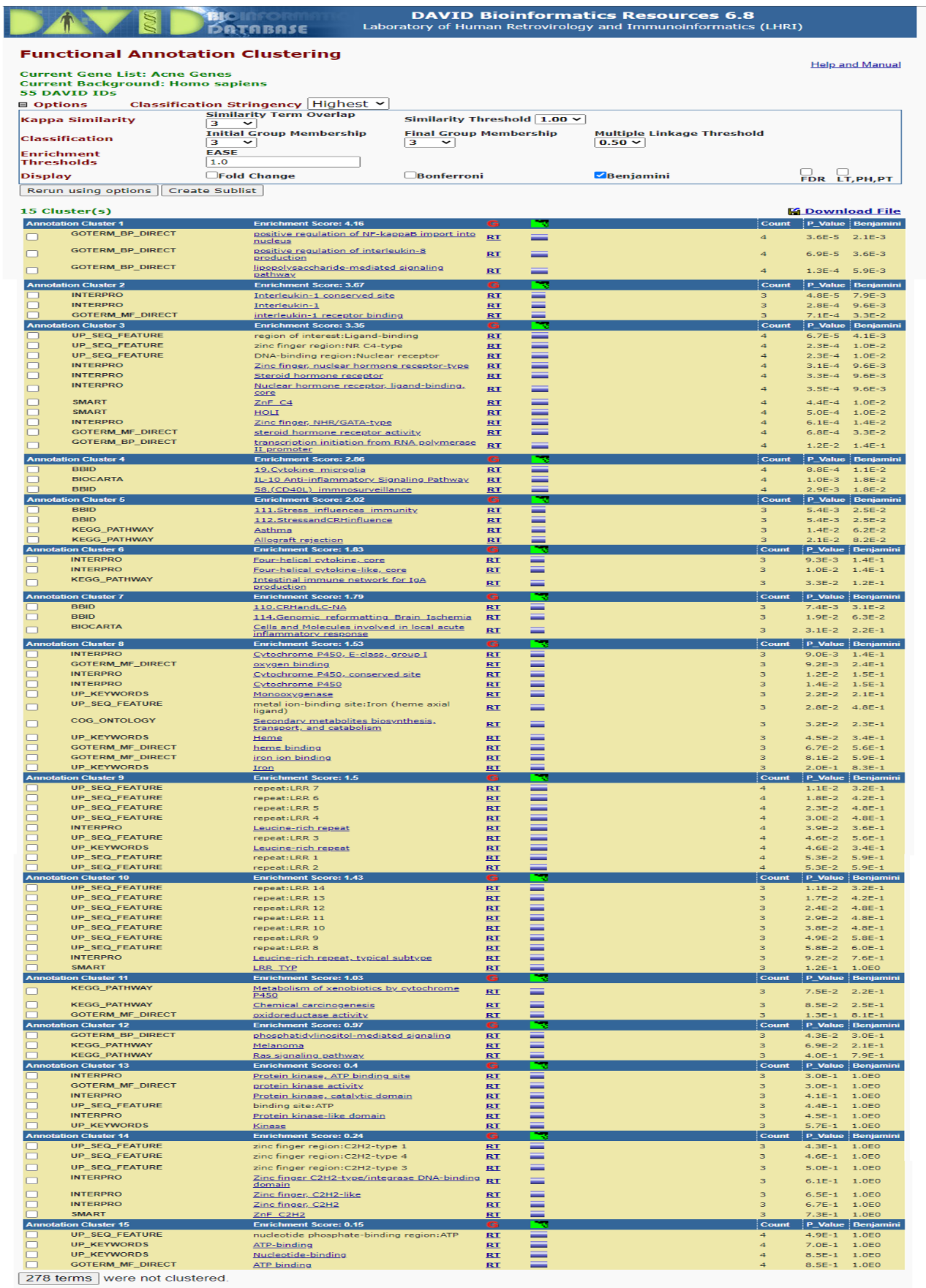


**Supplementary Figure S2** Gene ontology analysis/network analysis was performed using the online Database for Annotation, Visualization and Integrated Discovery (DAVID) v6.8 software. The software is available at <https://david.ncifcrf.gov/>. Analysis was performed with the highest classification stringency and other default settings for functional annotation clustering. A total of 15 clusters were detected.


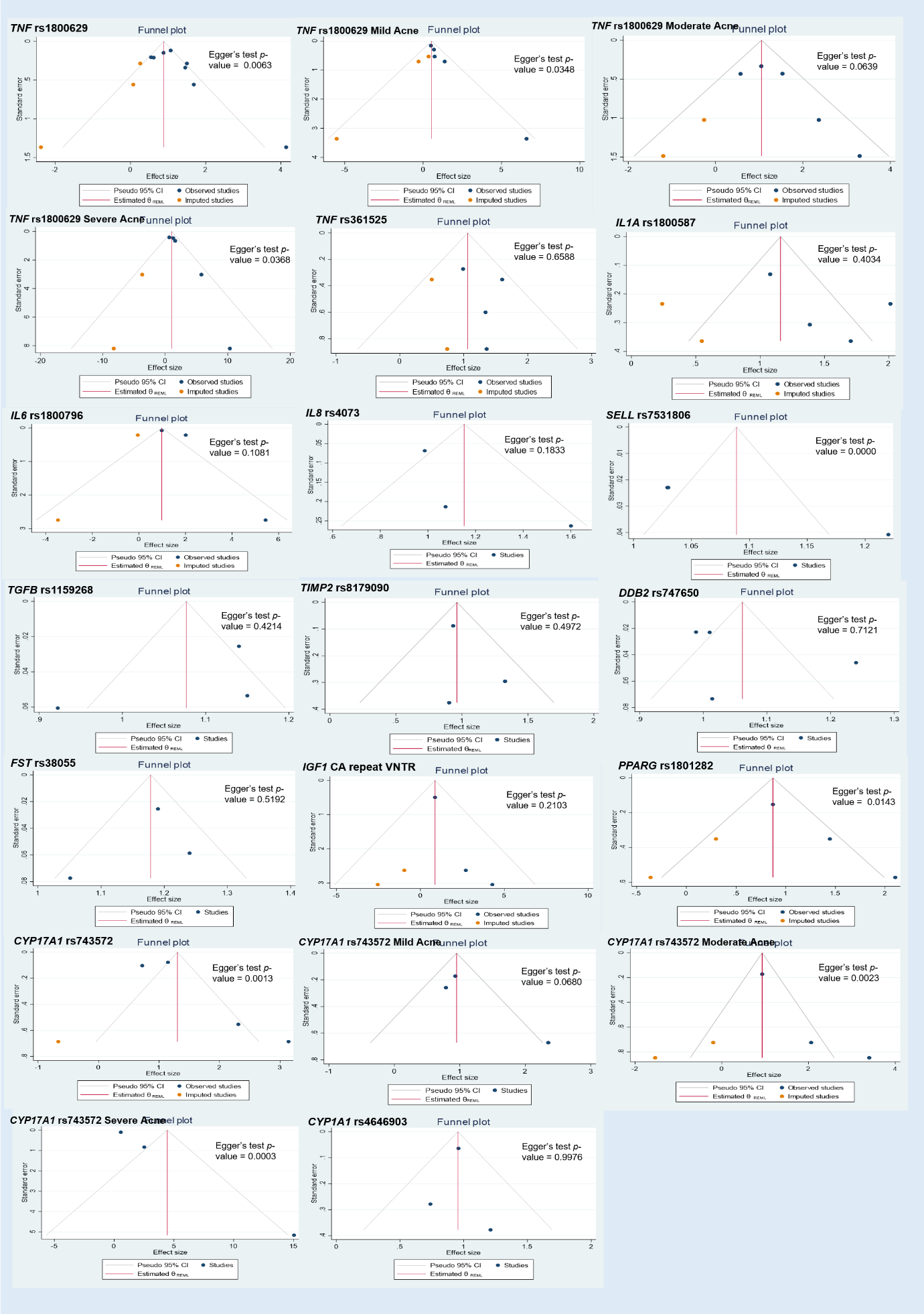


**Supplementary Figure S3** Assessment of publication bias using Begg’s funnel plots and

Egger’s test for the SNPs included in the meta-analysis.
